# Protein Language Model‐Driven Optimisation of Antimicrobial Peptide Pth‐Ca1 Against *Pectobacterium brasiliense* Using ESMFold‐Predicted Structures and the ESM‐3 Model

**DOI:** 10.1111/mpp.70250

**Published:** 2026-03-19

**Authors:** Linhui Song, Ge Zhang, Mengying Hua, Jingna Yuan, Jian Wu, Huan Wang, Fei Yan, Jiejun Peng

**Affiliations:** ^1^ State Key Laboratory for Quality and Safety of Agro‐Products, Key Laboratory of Biotechnology in Plant Protection of MARA, Zhejiang Key Laboratory of Green Plant Protection, Institute of Plant Virology Ningbo University Ningbo China; ^2^ Suzhou Academy of Agricultural Sciences Suzhou China

**Keywords:** antimicrobial peptides, ESM‐3, ESMFold, *Pectobacterium brasiliense*

## Abstract

The emergence of antimicrobial resistance (AMR) poses a significant threat to global health and food security. Antimicrobial peptides (AMPs), particularly those characterised by α‐helical structures, represent a promising alternative due to their broad‐spectrum activity and unique mechanisms of action. *Pectobacterium brasiliense* is a destructive bacterial pathogen affecting solanaceous crops but effective control measures remain insufficient. This study aims to optimise pseudothionin AMPs using AI‐based protein language models, ESM‐3 and ESMFold, to enhance their antibacterial efficacy. Using ESM‐3, we generated multiple analogs of Pth‐Ca1 with increased net charge, hydrophobicity and helical ratio. Among these, Design_1867 exhibited the strongest antibacterial activity against both *Escherichia coli* and *P. brasiliense* , with minimum inhibitory concentration (MIC) values of 31.25 μg/mL. Design_1867 was found to bind bacterial DNA and induce pore formation in bacterial membranes through a barrel‐stave mechanism, similar to the reference peptide alamethicin. Reverse transcription‐quantitative PCR analyses revealed the downregulation of key genes associated with membrane integrity and biofilm formation. In planta assays confirmed its efficacy and low cytotoxicity. This study demonstrates the successful application of ESM‐3 and ESMFold for the rational design of highly effective AMPs. Design_1867 exhibits potent antimicrobial activity against *P. brasiliense* with minimal toxicity, underscoring the potential of AI‐driven AMP optimisation for sustainable agricultural disease management.

## Graphical abstract

AI (ESM‐3/ESMFold) was used to design a potent antimicrobial peptide, Design_1867, derived from a plant defensin (Pth‐Ca1). Design_1867 exhibits enhanced antibacterial activity by forming barrel‐stave pores in bacterial membranes and binding genomic DNA, effectively protecting plants without significant cytotoxicity.

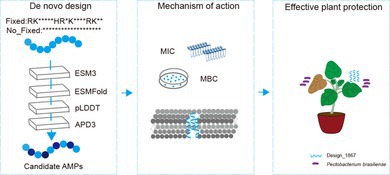

The relentless rise of antimicrobial resistance (AMR) represents one of the most pressing challenges to global public health and food security (Li et al. 2023). Antimicrobial peptides (AMPs), essential components of the innate immune system, offer a compelling alternative due to their broad‐spectrum activity and ability to circumvent common resistance mechanisms. Traditionally, AMP development has been largely empirical, relying on the isolation of peptides from natural sources followed by sequential amino acid substitutions—a process that is often time‐consuming and costly. Machine learning has revolutionised the discovery of AMPs by facilitating the de novo design of novel peptides and the development of analogs of peptides (Szymczak and Szczurek 2023). Specifically, enhancing the charge, hydrophobicity and helical ratio of α‐helical polypeptides through machine learning can significantly improve their antibacterial efficacy (Oliveira Júnior et al. 2025; Yeaman and Yount 2003; Wang et al. 2014, 2016; Wang 2013; Xi et al. 2016; Li et al. 2020; Krishnani et al. 2014).

Evolutionary Scale Modelling (ESM), a protein language model (PLM), infers the full atomic‐level three‐dimensional (3D) protein structure directly from primary sequences, distinguishing it from methods that utilise evolutionary information derived from multiple sequence alignments for structure prediction (Lin et al. 2023). The advanced Evolutionary Scale Modelling‐3 (ESM‐3) has emerged as a cutting‐edge multimodal generative language model that integrates reasoning across protein sequence, structure and function (Hayes et al. 2025). Capable of processing complex multimodal prompts, the upgraded ESM‐3 model demonstrates enhanced responsiveness to alignment information, thereby improving its predictive fidelity. We focused on *Pectobacterium brasiliense*, a globally distributed bacterial plant pathogen causing soft rot in a wide range of economically important crops (Duarte et al. 2004; de Werra et al. 2015), and pseudothionin from *Solanum tuberosum* (Pth‐St1), a 5 kDa defensin‐like polypeptide with activity against *Ralstonia solanacearum* , *Clavibacter michiganensis* subsp. *sepedonicus* and *Fusarium solani* (Moreno et al. 1994). A previous study showed Pth‐St1 has antimicrobial activity, but its application is limited at higher concentrations (Moreno et al. 1994).

We identified orthologous sequences of Pth‐St1 based on BLAST results for amino acid sequences (Table S1). The homologous protein of Pth‐St1 is highly conserved among mostly solanaceous crops, with the most positively charged amino acids being R (3), K (3) and H (1) in the defensin‐like protein of *Capsicum annuum* (Pth‐Ca1) (Table S1, Figure S1a). Analysis of amino acid characteristics revealed that both Pth‐St1 and Pth‐Ca1 have a hydrophobic ratio of 21%, with net charges of +2 and +5.25, respectively (Figure S1a). To identify the 3D structure of the peptides, AlphaFold 3 was used for structural prediction (Figure S1b). The 3D structures of Pth‐St1 and Pth‐Ca1 exhibited 8 and 12 helix residues, respectively (Figure S1a,b). To verify the antibacterial effects of Pth‐St1 and Pth‐Ca1, the MIC against *Escherichia coli* was determined using the doubling dilution method (Hammer et al. 1999). The antimicrobial activity of Pth‐Ca1 (MIC: 2 mg/mL) was superior to that of Pth‐St1 (MIC: > 2 mg/mL) (Figure S1c). This suggests that the net charge and number of helices may influence the antimicrobial efficiency of pseudothionins.

To enhance the net charge and ratio of helices, analogs of Pth‐Ca1 were designed by retaining positively charged amino acids or introducing random mutations that increase the net charge, hydrophobic amino acids and the formation of helical structures using the ESM‐3 model (Figure 1). Two mutation methods were employed: Fixed R, K, H of Pth‐Ca1 (RK_____HR_K____RK__) and No_Fixed (input: __________________). A total of 4093 peptides with No_Fixed mutations and 4471 peptides with Fixed mutations were generated. Subsequently, the ESMFold tool was used to predict the 3D structure of each peptide and to calculate its pLDDT value, as well as the number of helical amino acids (Figure 1). Based on the number of mutated amino acids, the number of helices and the pLDDT values, the top 13 mutant sequences from both the fixed and no_fixed mutant libraries are summarised in Table S2. This table includes the sequence ID, amino acid sequence, number of mutations, predicted accuracy (pLDDT) and the number of helical amino acids. The net charge and hydrophobic ratio were predicted using the APD3 tool (https://aps.unmc.edu/prediction) (Wang et al. 2026). From the filtering and screening results, eight sequences exhibiting a net charge greater than +5.25, a hydrophobic ratio exceeding 21%, and 17 helix residues were selected (Figure 2a).

**FIGURE 1:**
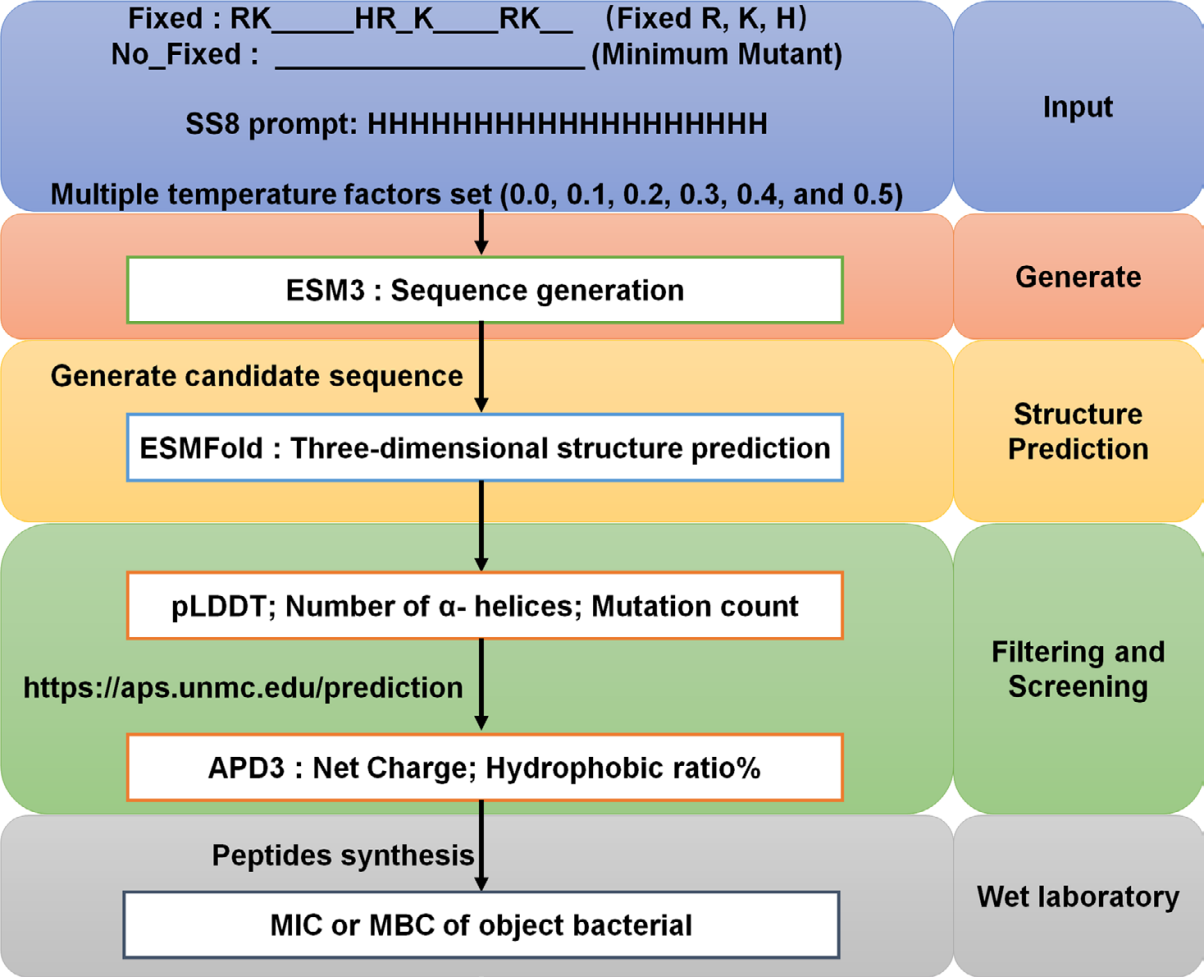
The workflow for de novo development of antimicrobial peptides (AMPs) via deep learning. The ESM‐3 model was used for No_Fixed and Fixed prediction to generate random mutant sequences that meet the requirements. The sequences underwent 3D structure prediction via ESMfold, and candidate sequences were screened based on pLDDT, number of α‐helix residues, and count of mutated amino acids. The screened sequences were further analysed for peptide sequence characteristics using the APD3 website. The peptides obtained after screening were subjected to MIC and MBC assays against target bacteria to validate their antimicrobial activity.

**FIGURE 2:**
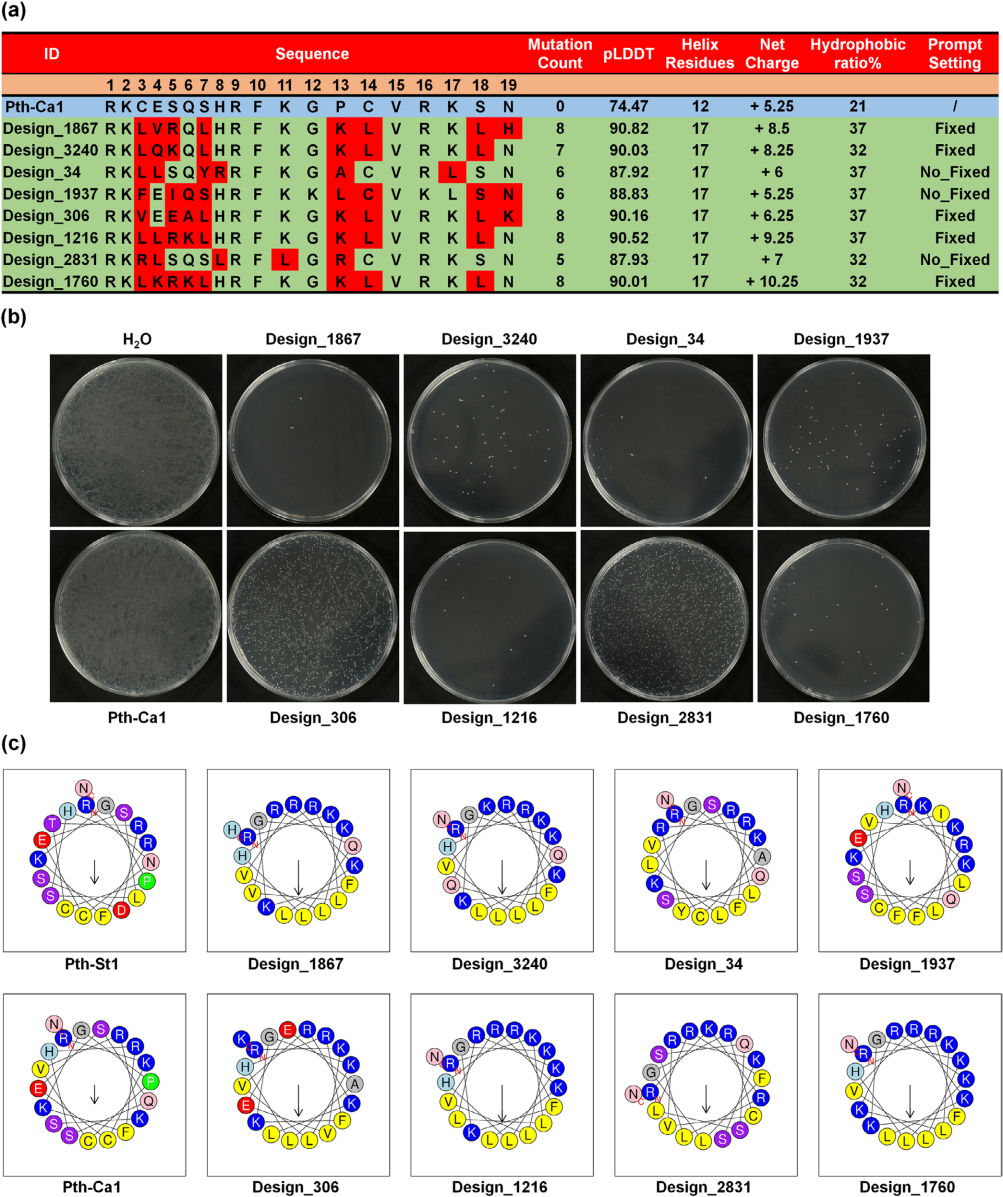
Characterization and antibacterial activity analysis of Pth‐Ca1 derivatives. (a) Pth‐Ca1‐derived peptides generated via ESM‐3 using No_Fixed and Fixed modes were screened, and candidate peptide sequences with a net charge greater than 5.25 and hydrophobicity greater than 21% were selected. (b) Chemically synthesised derivative peptides were mixed with *Escherichia coli* at a concentration of 200 μg/mL, and bacterial colony counts were used to determine the antibacterial activity of the derivatives. Design_1867 showed the best performance, with a colony count of 2.67 ± 0.58. (c) Helical wheel diagrams of Pth‐St1, Pth‐Ca1 and Pth‐Ca1 derivative peptides were analysed using HeliQuest. Yellow represents hydrophobic amino acids, blue represents positively charged amino acids, red represents negatively charged amino acids, and purple represents polar amino acids. Except for Pth‐Ca1, which did not form a hydrophobic face, all other peptides formed a hydrophobic face consisting of 2–5 amino acids. The hydrophobic face of Design_1867 is composed of the amino acids FLLLL.

To validate the structural stability of ESMFold predictions, we performed molecular dynamics (MD) simulations and compared the results with those from other structure prediction models (AlphaFold3, Chai‐1, Boltz‐2 and Protenix) for 25 candidate sequences. Notably, all five models independently predicted stable α‐helical conformations with highly consistent backbone geometries and secondary structure assignments (Figure S2). To complement these static structure predictions with physics‐based validation, we conducted all‐atom MD simulations (50 ns, explicit solvent, AMBER ff19SB force field) for the 25 candidate sequences. The MD trajectories demonstrated robust structural stability: all peptides maintained stable α‐helical conformations throughout the simulations, with low backbone RMSD values plateauing below 0.4 Å. Per‐residue secondary structure analysis (Figures [Link], [Link], [Link], heatmaps) revealed that the core helical regions (residues 2–17) exhibited greater than 90% helical occupancy, whereas only the C‐terminal residues (18–19) showed the expected flexibility typical of helix termini. Importantly, no unfolding events or transitions to random coil conformations were observed in any of the 25 simulations, confirming that the predicted helical structures represent thermodynamically stable conformations in aqueous solution (Figures S3 and S4, Table S3).

To evaluate the antimicrobial activities of Pth‐Ca1 derivatives, peptides at a concentration of 200 μg/mL were added to *E. coli* . The results indicated that Design_1867 (RKLVRQLHRFKGKLVRKLH) exhibited the best antimicrobial activity among the eight Pth‐Ca1 derivatives (Figure 2b, Table S4). To further investigate the sequence characteristics of the Pth‐Ca1‐derived peptides, HeliQuest was used to analyse the composition of their helical wheel projections (Gautier et al. 2008). Except for Pth‐Ca1, which did not form a hydrophobic face, all other peptides formed one hydrophobic face composed of 2–5 hydrophobic amino acids (Figure 2c; Table S4). The derivative peptides with a hydrophobic face consisting of FLLLL exhibited colony counts ranging from 2.67 to 76.40. The derivative peptides containing the negatively charged amino acid (E) (Design_306 and Design_1937) showed colony counts of > 200 and 74.33 ± 16.50, respectively. Derivatives with a hydrophobicity ratio below 0.1 (Design_3240), a net charge less than 6 (Design_1937), or fewer than 5 hydrophobic face amino acids (Design_2831) all exhibited colony counts greater than 74.33. These results suggest that peptides possessing a hydrophobic face, characterised by at least three consecutive hydrophobic amino acids, a Hyd (the mean hydrophobicity) value greater than 0.1, and an HMom (the mean amphipathic moment) value exceeding 0.5, exhibit enhanced activity. Conversely, the presence of negatively charged residues (e.g., glutamic acid) or a Hyd value below 0.1 diminishes efficacy.

Among these, Design_1867 exhibited the strongest antimicrobial activity against *E. coli* . To further verify the antibacterial effect of Design_1867, the MIC against *E. coli* was determined using the doubling dilution method. The results showed that 31.25 μg/mL of Design_1867 resulted in the inflection phenomenon of MIC. These findings suggest that Design_1867 has greater efficacy than Pth‐Ca1 and Pth‐St1 (Figures S1a and S5a). Subsequently, the antibacterial activity of Design_1867 was assessed at different growth stages based on the antibacterial curve. Compared to the phosphate‐buffered saline (PBS) control, treatment with 8× MIC (250 μg/mL) of Design_1867 completely inhibited the growth of *E. coli* after 12 h (Figure S2b). To investigate the binding affinity of Design_1867 to *E. coli* genomic DNA (gDNA), various concentrations of Design_1867, Pth‐Ca1 and Pth‐St1 were incubated with *E. coli* gDNA in a 37°C water bath for 30 min, using A19 (AAAAAAAAAAAAAAAAAAA) as a control. Following incubation, the samples were analysed using nucleic acid gel electrophoresis. The results indicated that at a 5:1 ratio (Design_1867: gDNA), the concentration of Design_1867 led to a progressive decrease in the intensity of gDNA bands, supporting the conclusion that Design_1867 binds to gDNA. Additionally, at a 10:1 ratio of Pth‐Ca1 to gDNA, binding was observed; however, no binding was detected for A19 and Pth‐St1, even at a 5:1 ratio of peptides to gDNA (Figure S5c).

Design_1867 was also highly effective against *P. brasiliense* , with an MIC of 31.25 μg/mL and an MBC of 62.5 μg/mL (Figure 3a,b). To analyse the effect of Design_1867 on *P. brasiliense* , scanning electron microscopy (SEM) was employed to observe Design_1867‐treated *P. brasiliense* at 0‐ and 6‐h post‐treatment (hpt). Scanning electron microscopy observations revealed that at 6 hpt Design_1867 induced the formation of one to three circular pores with distinct, sharp edges on the bacterial surface. The damage was localised, with phospholipids displaced solely at the pore sites, whereas the surrounding membrane structure remained intact and smooth. The pores appeared as clean, neatly punched holes in the membrane (Figure 3c). After treatment with Design_1867 for 6 h, 47.0% of the bacteria exhibited perforation, which was significantly higher than the control (Figure 3d). These findings suggest that the mechanism of action of Design_1867 follows the barrel‐stave model (Shai 1999). To further confirm the mechanism of action of Design_1867, an AMP with known mechanism consistent with the barrel‐stave model, specifically alamethicin (Laver 1994), was selected as reference subject and observed under SEM at 0 and 6 hpt. The results indicated that the morphological changes induced by Design_1867 treatment were similar to those caused by alamethicin (Figure 3c). In addition, we conducted a concentration‐gradient assay of Design_1867 to further validate its membrane‐disrupting mechanism. A β‐galactosidase leakage assay was performed, revealing a clear, concentration‐dependent increase in the ONPG hydrolysis signal upon treatment with the AMP (Figure 3e). To investigate the molecular mechanisms underlying the response of *P. brasiliense* to Design_1867 and alamethicin, reverse transcription‐quantitative real‐time PCR (RT‐qPCR) was employed to assess the expression levels of genes related to substance diffusion, superoxide formation, lipopolysaccharide (LPS) layer formation and biofilm formation. The expression of *bolA* (a biofilm‐related gene), outer membrane porin F (*ompF*), *robA* (a reactive oxygen species‐related gene) and *lptF* (involved in the translocation of LPS from the inner membrane to the outer membrane) was measured (Table S5). The downregulation of *ompF* induced by Design_1867 and alamethicin (73.3% and 84.3% decreases, respectively) indicated destabilisation of the outer membrane. Additionally, both Design_1867 and alamethicin led to downregulation of *lptF* (96.0% and 94.0% decreases, respectively), *robA* (94.0% and 92.7% decreases, respectively) and *bolA* (73.3% and 87.3% decreases, respectively). These findings suggest that Design_1867 operates in a manner analogous to the barrel‐stave model.

**FIGURE 3:**
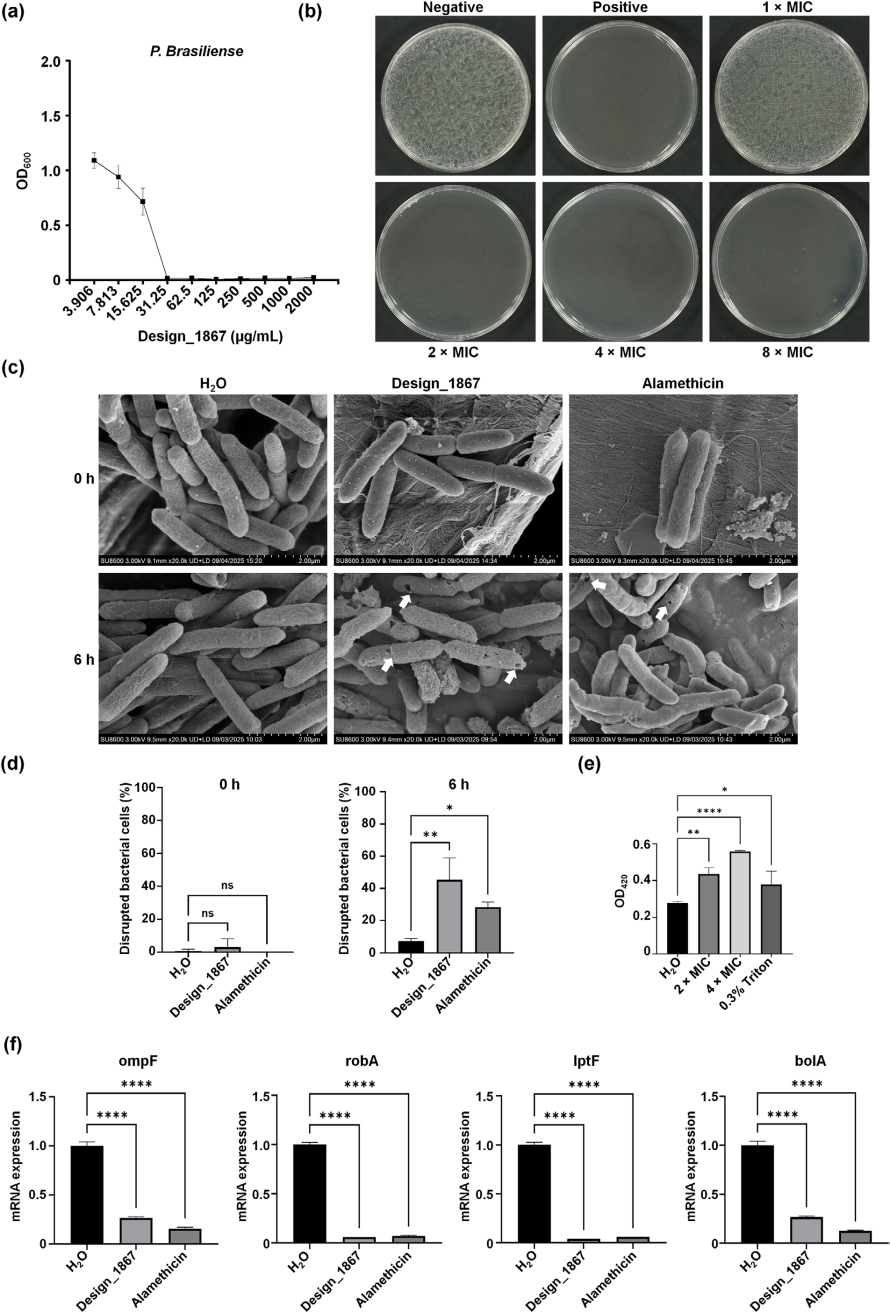
Investigation of the antibacterial activity of Design_1867 against *Pectobacterium brasiliense*. (a) The MIC of Design_1867 against *P. brasiliense* was determined at concentrations ranging from 3.906 to 2000 μg/mL. Design_1867 at 31.25 μg/mL exhibited significant antibacterial effects. (b) The MBC of Design_1867 against *P. brasiliense* was validated using 1×–8× MIC. The MBC of Design_1867 for *P. brasiliense* was determined to be 2× MIC (62.5 μg/mL). (c) Morphological changes of *P. brasiliense* after co‐culture with the peptides were observed using scanning electron microscopy. *P. brasiliense* at a concentration of 1 × 10^9^ CFU/mL (500 μL) was mixed with 500 μL of Design_1867 at a concentration of 2× MIC (62.5 μg/mL) and treated for 0 and 6 h. Alamethicin at 2× MIC (2000 μg/mL) was used as a positive control (known to act via a barrel‐stave pore mechanism). There were no significant morphological changes in *P. brasiliense* treated with Design_1867, water, or alamethicin at 0 h. After 6 h of treatment, both Design_1867 and alamethicin induced the formation of well‐defined pore structures with smooth edges in *P. brasiliense* . (d) The percentages of bacteria with membrane perforation were quantified for Design_1867, water, or alamethicin at 0 and 6 h. No significant difference in perforation rate was observed at 0 h. At the 6‐h time point, the percentages for both Design_1867 and alamethicin were significantly higher than the control. *p < 0.05, **p < 0.01, ^ns^ *p* > 0.05. (e) Design_1867 was added to final concentrations of 0× MIC, 2× MIC and 4× MIC, with 0.3% (v/v) Triton X‐100 included as a positive control. ONPG (*O*‐nitrophenyl‐β‐D‐galactopyranoside) was added to each well to monitor bacterial intracellular content leakage, followed by incubation at 37°C. Absorbance was measured at 420 nm using a microplate reader. The increase in Design_1867 concentration led to a higher degree of ONPG hydrolysis, as indicated by the elevated A_420_ readings, demonstrating that higher peptide concentrations resulted in greater leakage of intracellular components (*p < 0.05, **p < 0.01, ****p < 0.0001). (f) The expression levels of four genes (*ompF*, *robA*, *lptF* and *bolA*) were determined by reverse transcription‐quantitative PCR after 6 h of treatment, using 16S rRNA as the internal reference gene. Three biological replicates were included and one representative is shown (****p < 0.0001). Compared to the control, *ompF*, *robA*, *bolA* and *lptF* were downregulated in the Design_1867 and alamethicin treatment groups.

To confirm the cytotoxicity and antibacterial efficacy of Design_1867 in vivo, the cytotoxic effects of Design_1867 were evaluated at concentrations ranging from 0 to 500 μg/mL against HK‐2 renal proximal tubular cells in a CCK‐8 assay, using water as a control and performing five experimental replicates. The results demonstrated that 500 μg/mL of Design_1867 had no significant toxicity to the cell line (Table S6). To further evaluate the cytotoxicity of Design_1867 in plants, 2× MIC of Design_1867 was injected into the leaves of *Nicotiana benthamiana*, using water as a control. At 24 h post‐inoculation (hpi), no significant necrotic symptoms were observed on the leaves treated with 2× MIC of Design_1867 (Figure 4a). To test the inhibitory effect of Design_1867 against *P. brasiliense* in plants, a mixture of *P. brasiliense* and 2× MIC of Design_1867 was co‐injected into *N. benthamiana* leaves, again using water as a control. At 24 hpi, obvious necrosis was observed on the control side of the leaves, whereas the side injected with the mixture of Design_1867 and *P. brasiliense* had no significant necrotic symptoms (Figure 4b,c). We also performed a direct comparison of the in vivo antimicrobial activity between Design_1867 and the wild‐type Pth‐Ca1 in planta. The results demonstrated that Design_1867 exhibited enhanced antibacterial efficacy in the plant system compared with the wild‐type peptide (Figure S6). Previous studies have shown that functional peptides can inhibit root growth (Hou et al. 2021). Therefore, we conducted root growth inhibition assays as well as seed germination assays to more comprehensively evaluate the potential phytotoxicity of Design_1867. The results showed no significant differences in root length or seed germination rate between Design_1867‐treated plants and the control group, indicating that Design_1867 does not adversely affect early plant growth under the tested conditions (Figure S7). Thus, Design_1867 exhibits no significant cytotoxicity to plant cells and effectively inhibits *P. brasiliense* infection in vivo.

**FIGURE 4:**
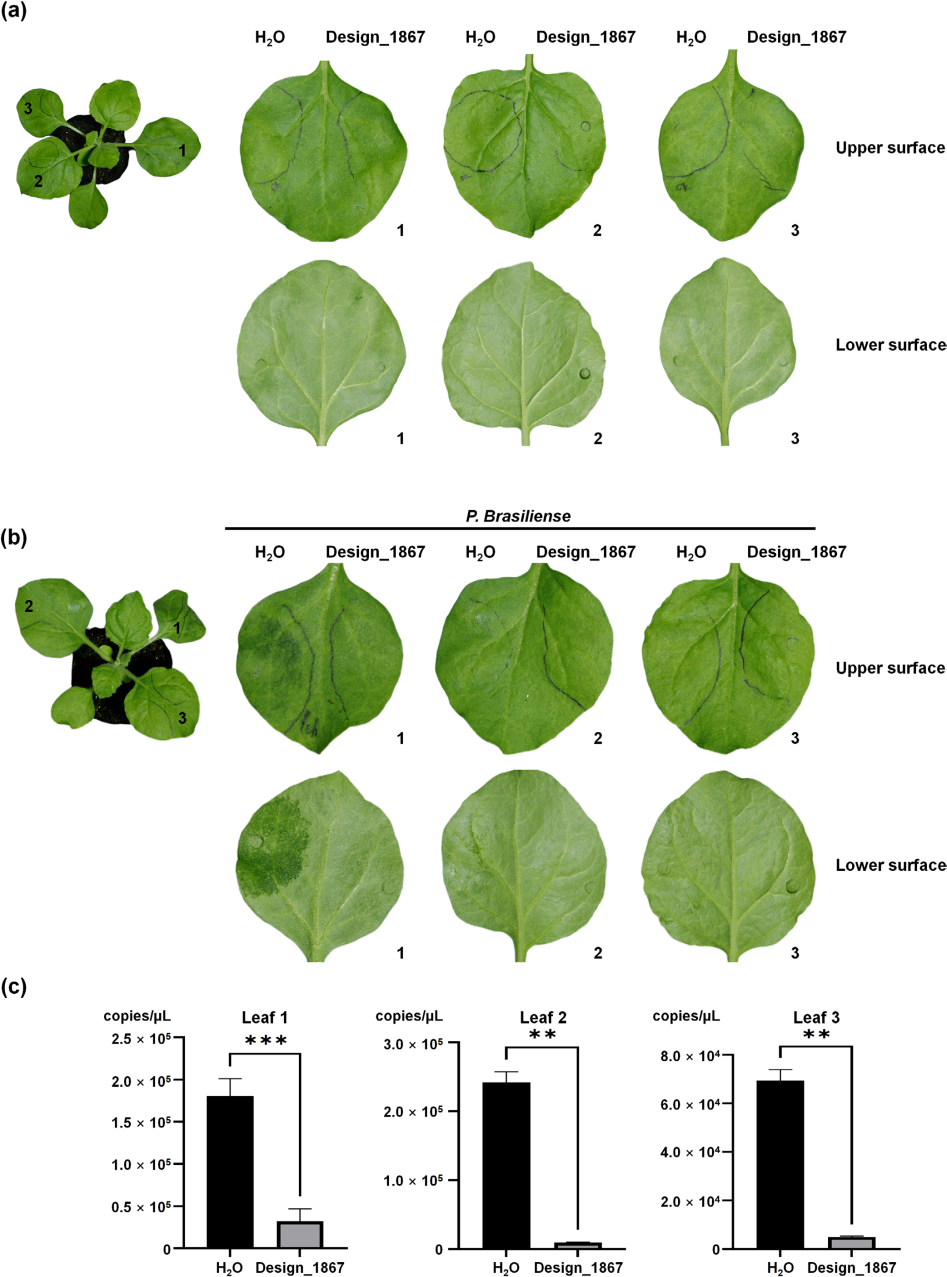
Investigation of the inhibitory effect of Design_1867 against *Pectobacterium brasiliense* in *Nicotiana benthamiana*. (a) Toxicity study on *N. benthamiana* leaves injected with 2× MIC (62.5 μg/mL) of Design_1867 or water for 6 h. No significant necrotic lesions were observed in leaves injected with Design_1867. (b) *P. brasiliense* bacterial suspension (OD_600_ = 0.1) was mixed with an equal volume of Design_1867 (62.5 μg/mL) and injected into *N. benthamiana* leaves for 6 h. The control side exhibited water‐soaked rot, whereas the Design_1867‐treated side showed no or only mild rot. (c) Absolute quantitative PCR detection of *P. brasiliense* copy numbers in control and Design_1867‐treated leaves indicated that the bacterial load in the Design_1867‐treated group was significantly lower than that in the control group (three biological replicates were included and one representative is shown, **p < 0.01, ***p < 0.001).

Currently, the prediction and redesign of AMPs primarily follow two major approaches: one based on peptide structure analysis and the other relying on machine learning using AMP databases. Structure‐based methods include AlphaFold (Jumper et al. 2021), which uses multiple sequence alignment training with a large number of experimentally determined structures from the Protein Data Bank (PDB). Conversely, ESMfold (Hayes et al. 2025; Lin et al. 2023) and RFdiffusion (Vázquez Torres et al. 2024) focus on the direct inference of full atomic‐level protein structures from primary sequences. Initially, AI‐based predictions predominantly relied on natural AMPs sourced from the APD3 for training and prediction. In recent years, predictive efforts have increasingly incorporated hypothetical negative datasets obtained from UniProt, alongside positive datasets integrated from databases such as the APD that include more synthetic peptides (Yao et al. 2025). In this study, we employed ESM‐3 for structure‐directed optimisation and used ESMfold for structural prediction. Candidate derivatives of Pth‐Ca1 were screened based on three criteria: α‐helical content, net charge and hydrophobicity. Although the accuracy of this method is constrained by the antibacterial mechanisms of α‐helical AMPs, it has been demonstrated that more precise constraints can lead to enhanced antimicrobial activity in the derived peptides.

Amphipathicity is a hallmark structural feature of α‐helical AMPs. The hydrophobic and hydrophilic faces are key structural features used to evaluate amphipathicity. Formation of a hydrophobic face in amphipathic α‐helices is widely considered to facilitate peptide interaction with lipid membranes (Tossi et al. 2000). Using the HeliQuest discriminant factor (*D*), when the predicted helix ratio exceeds 80%, the *D* value can be calculated as: 𝐷 = 0.944 × 𝜇𝐻 + 0.33 × *z* (Gautier et al. 2008). According to the *D* value, helices can be classified as follows: *D* < 0.68: helix; 0.68 ≤ *D* ≤ 1.34: possible lipid‐binding helix; *D* > 1.34: lipid‐binding helix. On this basis, ESMFold and HeliQuest predicted that the eight Pth‐Ca1 derivatives are lipid‐binding helices (Table S7). Another factor that contributes to enhanced antibacterial activity is the presence of positively charged residues on the hydrophilic face, which facilitate electrostatic interactions with negatively charged bacterial membranes. Therefore, peptides lacking a hydrophilic face (Design_2831 and Design_1937) or containing a negatively charged hydrophilic face (Design_306) had weaker antibacterial activity than Design_1867 (Table S4). Furthermore, having a Hyd below 0.1 was also disadvantageous for improving antibacterial activity (Design_1760, Design_3240). Overall, these results indicate that ESM‐3 redesign of Pth‐Ca1 increases net charge, helicity and hydrophobicity while maintaining a balanced optimisation of amphipathic structural features including Hyd, HMom and the organisation of the hydrophobic and hydrophilic faces. This coordinated optimisation enhances membrane‐binding capability and ultimately leads to increased antibacterial activity.

In conclusion, this study successfully demonstrates the application of ESM‐3 and ESMFold for the de novo design and optimisation of AMPs. By rationally enhancing net charge, hydrophobicity and helical content, we developed Design_1867, a potent peptide derivative that exhibits effectiveness against *E. coli* and the phytopathogen *P. brasiliense* . This peptide is proposed to operate through a barrel‐stave mechanism and shows no significant cytotoxicity. This AI‐driven approach offers a powerful strategy for accelerating the discovery of effective AMPs to combat resistant bacteria in agricultural and other contexts.

## Author Contributions

**Linhui Song:** methodology, data curation, writing – original draft preparation (equal). **Ge Zhang:** methodology, investigation, software; **Mengying Hua:** methodology. **Jingna Yuan:** methodology. **Jian Wu:** writing – review and editing. **Huan Wang:** conceptualization, writing – review and editing (equal). **Fei Yan:** conceptualization, writing – review and editing (equal). **Jiejun Peng:** conceptualization, data curation, software, writing – original draft preparation (lead); writing – review and editing (lead).

## Conflicts of Interest

The research methods and technical details presented in this study are currently the subject of a patent application.

## Supporting information


**Figure S1:** Characteristics of Pth‐St1 and Pth‐Ca1.

**Figure S2:** Multi‐model structural prediction consensus validates designed peptide architectures.

**Figure S3:** Molecular dynamics simulations confirm the thermodynamic stability of the fixed‐designed helices shown in Figure S2a.

**Figure S4:** Molecular dynamics simulations confirm the thermodynamic stability of the No_Fixed‐designed helices shown in Figure S2b.

**Figure S5:** Investigation of the antibacterial effect and DNA‐binding characteristics of Design_1867 against *Escherichia coli* ATCC25922.

**Figure S6:** Comparison of the inhibitory effect of Pth‐Ca1 and Design_1867 against *Pectobacterium brasiliense* in *Nicotiana benthamiana*.

**Figure S7:** Phytotoxicity assessment of Design_1867 in plants.

**File S1:** Experimental procedures.

**Table S1:** Homologous genes of Pth‐St1.

**Table S2:** Analysis of fixed and No_Fixed mutations in Pth‐Ca1.

**Table S3:** Structural comparison between ESM‐3 generated structures and ESMFold predictions.

**Table S4:** Characterisation and antimicrobial activity of Pth‐Ca1 and its derivatives.

**Table S5:** Primers used in this study.

**Table S6:** Cytotoxicity of the designed peptide 1867.

**Table S7:** Calculation of the discriminant factor *D*.

## Data Availability

The data that supports the findings of this study are available in Supporting Information of this article.
