# Supplementary material for: Protein Language Model‐Driven Optimisation of Antimicrobial Peptide Pth‐Ca1 Against Pectobacterium brasiliense Using ESMFold‐Predicted Structures and the ESM‐3 Model
Source: Mol Plant Pathol. 2026 Mar 19;27(3):e70250. doi: 10.1111/mpp.70250 (PMC13097337; doi:10.1111/mpp.70250)
Supplement: Supplementary file 1 — Figure S1: Characteristics of Pth‐St1 and Pth‐Ca1. [file MPP-27-e70250-s001.docx]

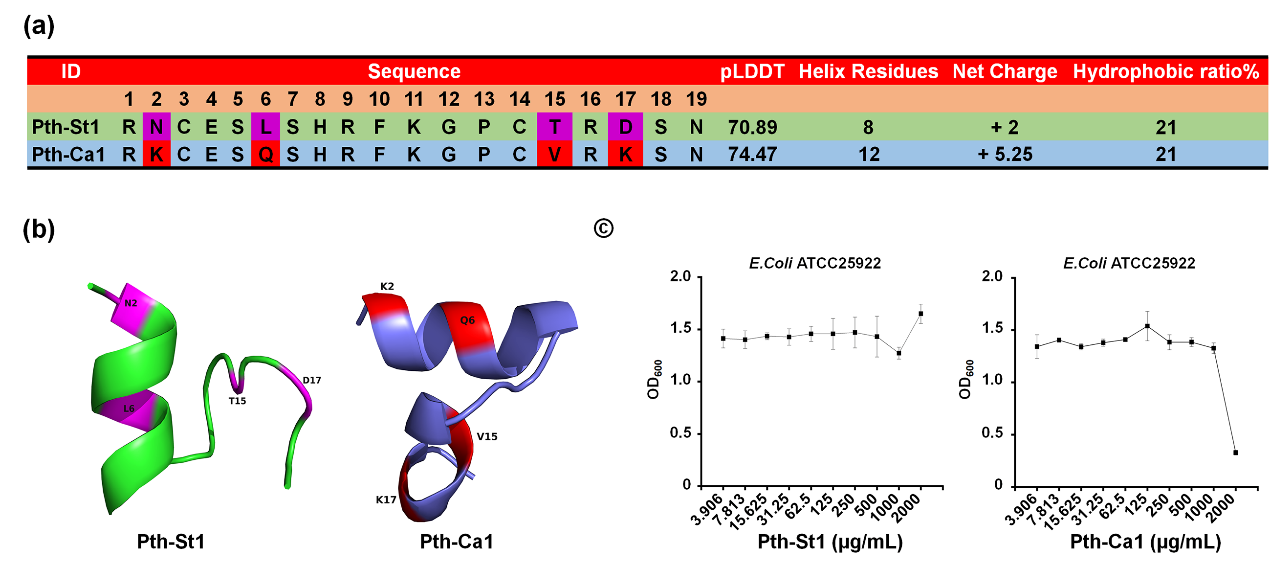


Figure S1. Characteristics of Pth-St1 and Pth-Ca1. (a) Comparison of amino acid sequences, pLDDT, number of helix residues, net charge, and percentage of hydrophobic amino acids between Pth-St1 and Pth-Ca1; (b) Protein structure prediction of Pth-St1 and Pth-Ca1 using AlphaFold3, showing that Pth-St1 has fewer helix residues than Pth-Ca1; (c) Minimum inhibitory concentration (MIC) assay of Pth-St1 and Pth-Ca1, where the MIC of Pth-Ca1 is 2000 μg/mL, and the MIC of Pth-St1 is >2000 μg/mL.
