# Supplementary material for: Protein Language Model‐Driven Optimisation of Antimicrobial Peptide Pth‐Ca1 Against Pectobacterium brasiliense Using ESMFold‐Predicted Structures and the ESM‐3 Model
Source: Mol Plant Pathol. 2026 Mar 19;27(3):e70250. doi: 10.1111/mpp.70250 (PMC13097337; doi:10.1111/mpp.70250)
Supplement: Supplementary file 2 — Figure S2: Multi‐model structural prediction consensus validates designed peptide architectures. [file MPP-27-e70250-s008.docx]

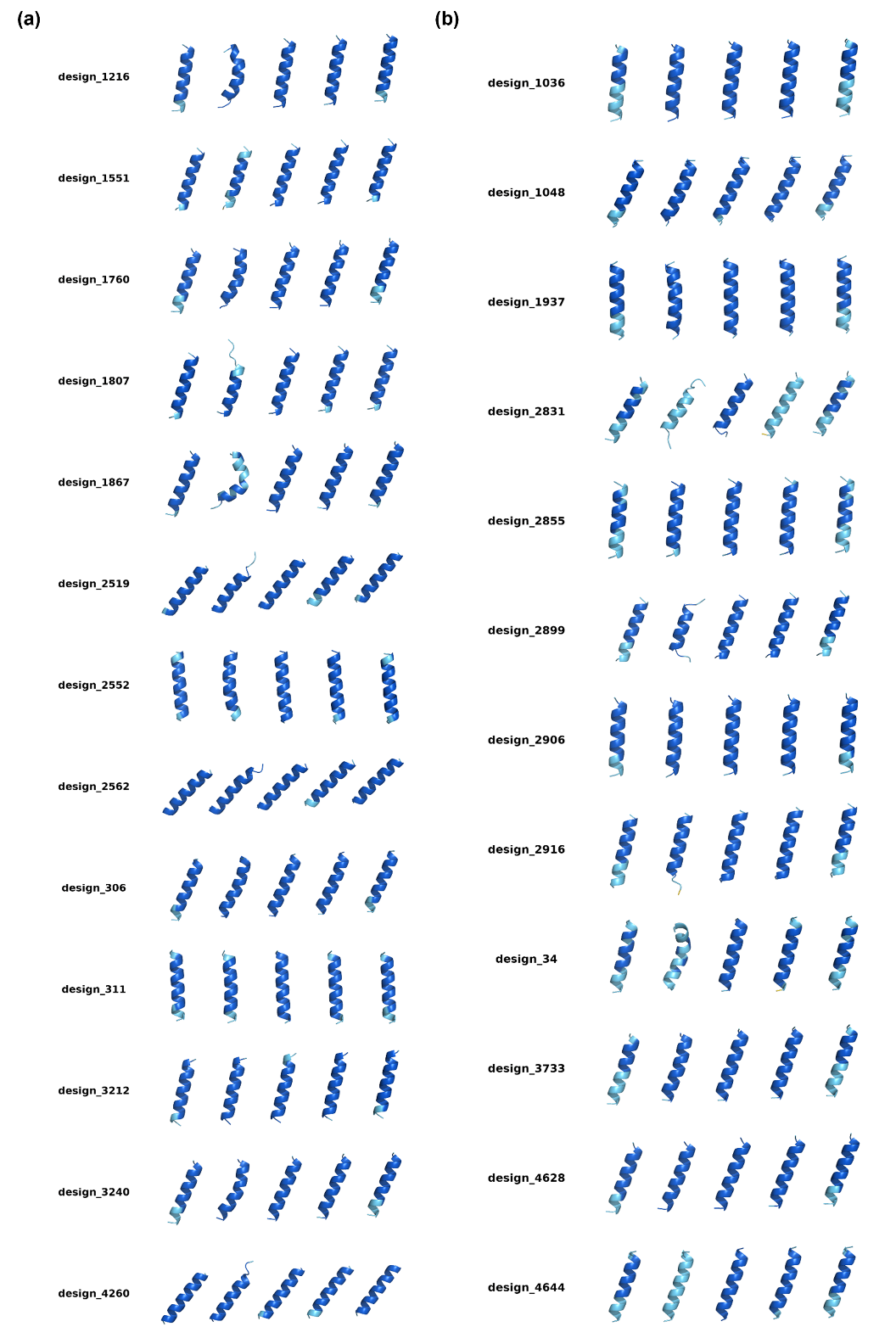


Figure S2. Multi-model structural prediction consensus validates designed peptide architectures. Structural overlays and secondary structure assignments from five independent prediction models (ESMFold, AlphaFold3, Chai-1, Boltz-2, Protenix) for representative fixed (a) and No_Fixed (b) candidates. Structures are colored by per-residue confidence (pLDDT): dark blue (>90, very high), light blue (70-90, confident), yellow (50-70, low), orange (<50, very low).
