# Supplementary material for: Protein Language Model‐Driven Optimisation of Antimicrobial Peptide Pth‐Ca1 Against Pectobacterium brasiliense Using ESMFold‐Predicted Structures and the ESM‐3 Model
Source: Mol Plant Pathol. 2026 Mar 19;27(3):e70250. doi: 10.1111/mpp.70250 (PMC13097337; doi:10.1111/mpp.70250)
Supplement: Supplementary file 3 — Figure S3: Molecular dynamics simulations confirm the thermodynamic stability of the fixed‐designed helices shown in Figure S2a. [file MPP-27-e70250-s005.docx]

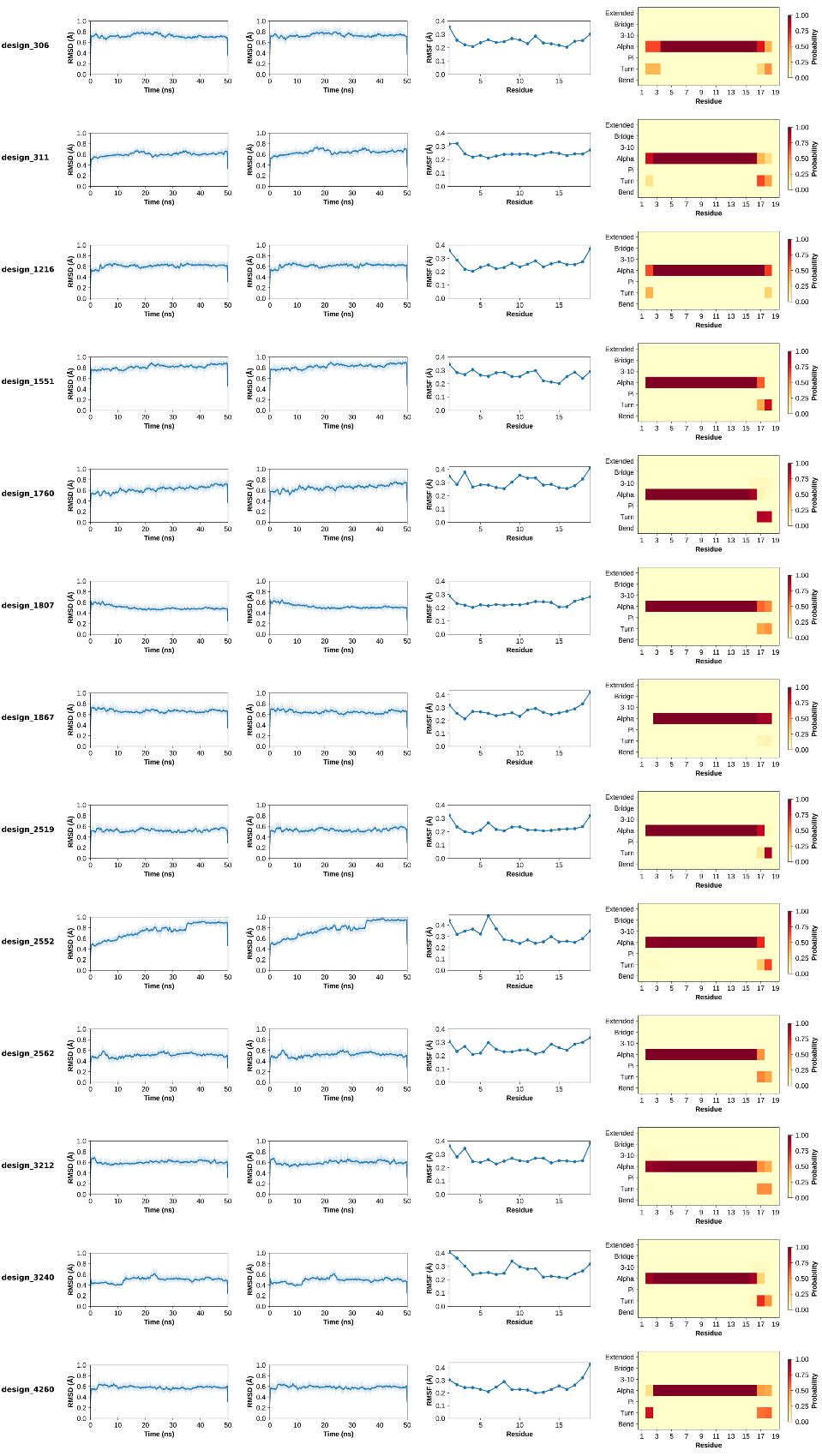


Figure S3. Molecular dynamics simulations confirm the thermodynamic stability of the Fixed-designed helices shown in Figure S2a. (Left) RMSD trajectories of 13 candidates over 50 ns. (Right) Per-residue secondary structure occupancy heatmaps showing sustained helical content throughout simulations.
