# Supplementary material for: Protein Language Model‐Driven Optimisation of Antimicrobial Peptide Pth‐Ca1 Against Pectobacterium brasiliense Using ESMFold‐Predicted Structures and the ESM‐3 Model
Source: Mol Plant Pathol. 2026 Mar 19;27(3):e70250. doi: 10.1111/mpp.70250 (PMC13097337; doi:10.1111/mpp.70250)
Supplement: Supplementary file 4 — Figure S4: Molecular dynamics simulations confirm the thermodynamic stability of the No_Fixed‐designed helices shown in Figure S2b. [file MPP-27-e70250-s012.docx]

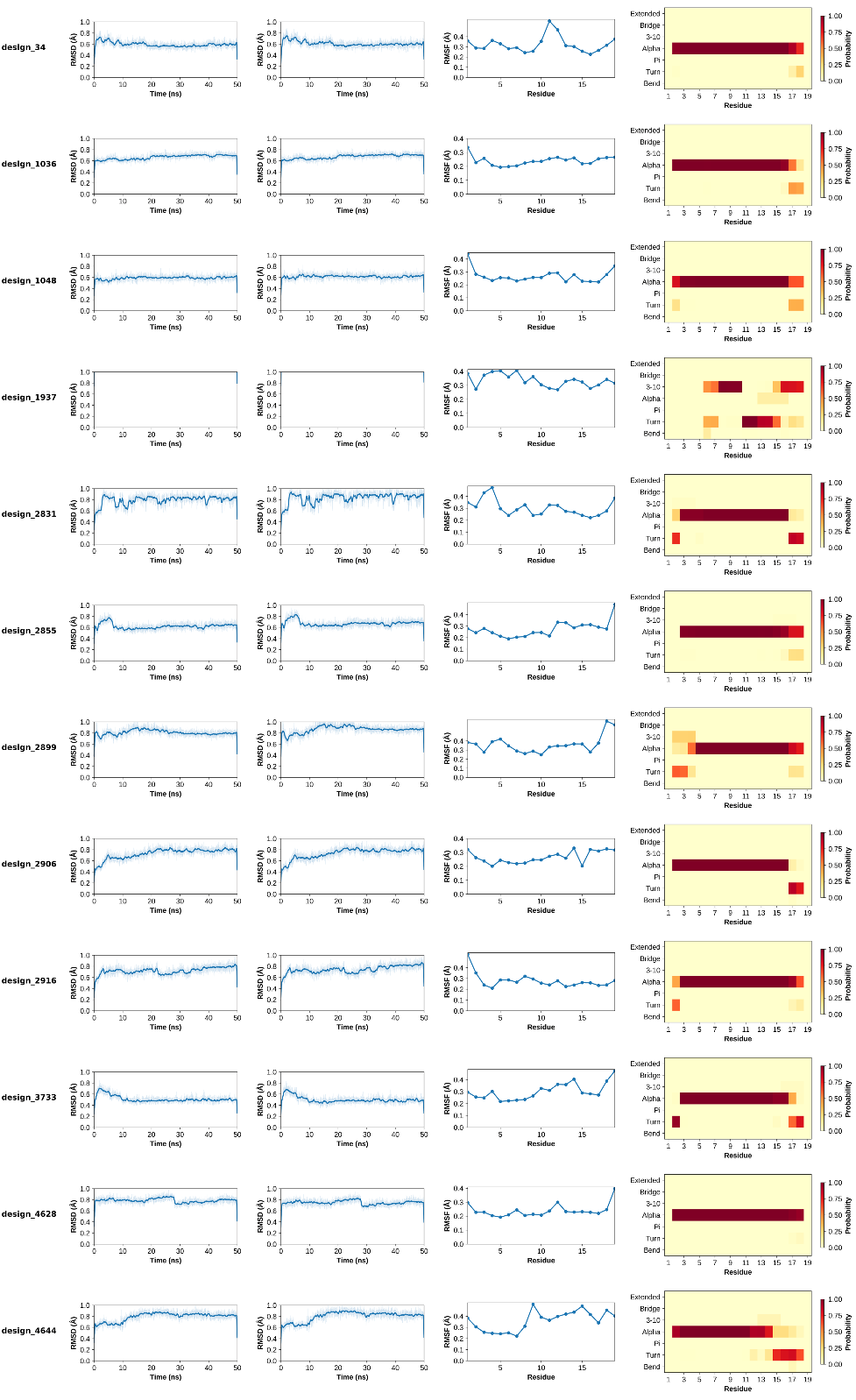


Figure S4. Molecular dynamics simulations confirm the thermodynamic stability of the No_Fixed-designed helices shown in Figure S2b. (Left) RMSD trajectories of the remaining 12 candidates over 50 ns. (Right) Per-residue secondary structure occupancy heatmaps showing sustained helical content throughout simulations.
