# Supplementary material for: Protein Language Model‐Driven Optimisation of Antimicrobial Peptide Pth‐Ca1 Against Pectobacterium brasiliense Using ESMFold‐Predicted Structures and the ESM‐3 Model
Source: Mol Plant Pathol. 2026 Mar 19;27(3):e70250. doi: 10.1111/mpp.70250 (PMC13097337; doi:10.1111/mpp.70250)
Supplement: Supplementary file 5 — Figure S5: Investigation of the antibacterial effect and DNA‐binding characteristics of Design_1867 against Escherichia coli ATCC25922. [file MPP-27-e70250-s013.docx]

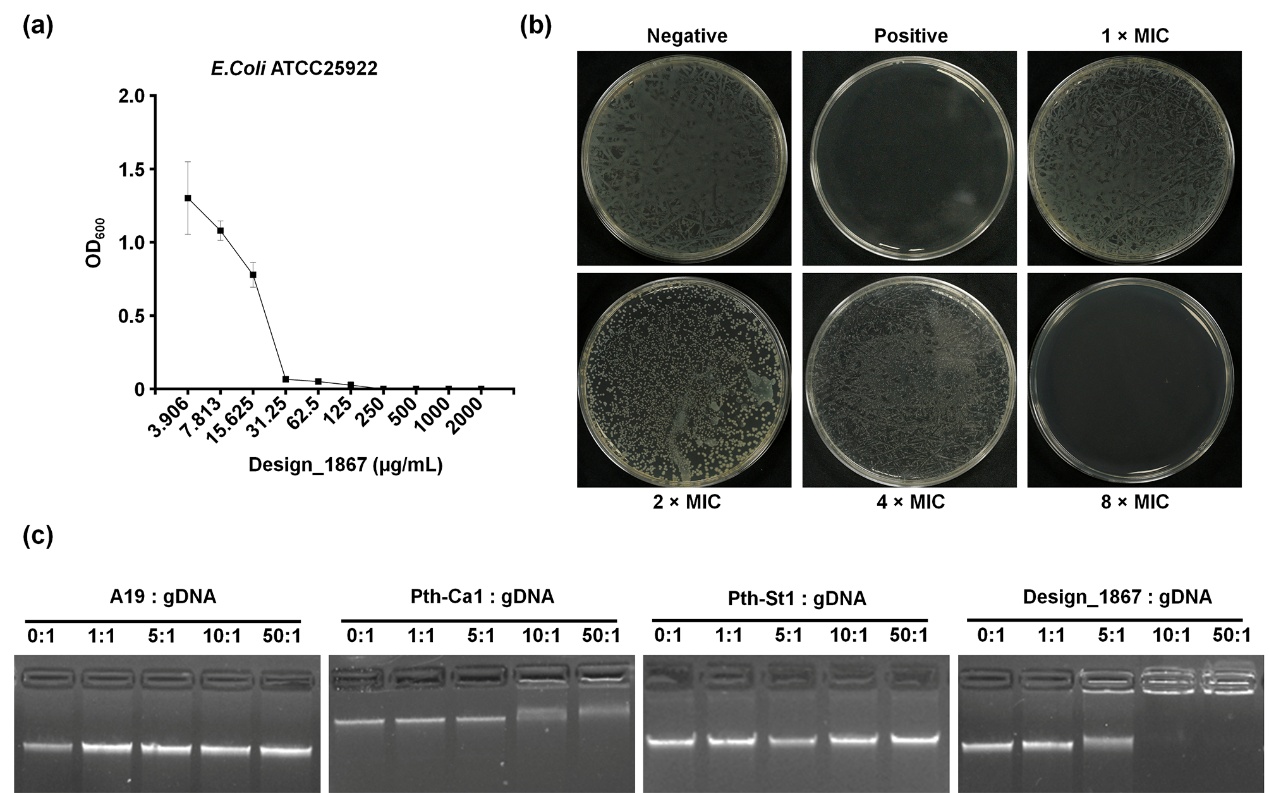


Figure S5. Investigation of the antibacterial effect and DNA-binding characteristics of Design_1867 against *E. coli* ATCC25922. (a) The MIC of Design_1867 against *E. coli* was determined at concentrations ranging from 3.906 to 2000 μg/mL. Design_1867 at 31.25 μg/mL exhibited significant antibacterial effects against *E. coli*; (b) The MBC of Design_1867 against *E. coli* ATCC25922 was validated using 1× to 8× MIC. The MBC of Design_1867 for *E. coli* was determined to be 8× MIC (250 μg/mL); (c) Gel electrophoresis was used to assess the binding efficiency of the peptide to genomic DNA at mass ratios (peptide: *E. coli* genomic DNA) of 0:1, 1:1, 5:1, 10:1, and 50:1. Design_1867 and Pth-Ca1 bound to gDNA at ratios of 5:1 and 10:1, respectively, causing retardation of gDNA migration. Design_1867 completely retarded gDNA migration at a ratio of 10:1.
