# Supplementary material for: Protein Language Model‐Driven Optimisation of Antimicrobial Peptide Pth‐Ca1 Against Pectobacterium brasiliense Using ESMFold‐Predicted Structures and the ESM‐3 Model
Source: Mol Plant Pathol. 2026 Mar 19;27(3):e70250. doi: 10.1111/mpp.70250 (PMC13097337; doi:10.1111/mpp.70250)
Supplement: Supplementary file 6 — Figure S6: Comparison of the inhibitory effect of Pth‐Ca1 and Design_1867 against Pectobacterium brasiliense in Nicotiana benthamiana. [file MPP-27-e70250-s010.docx]

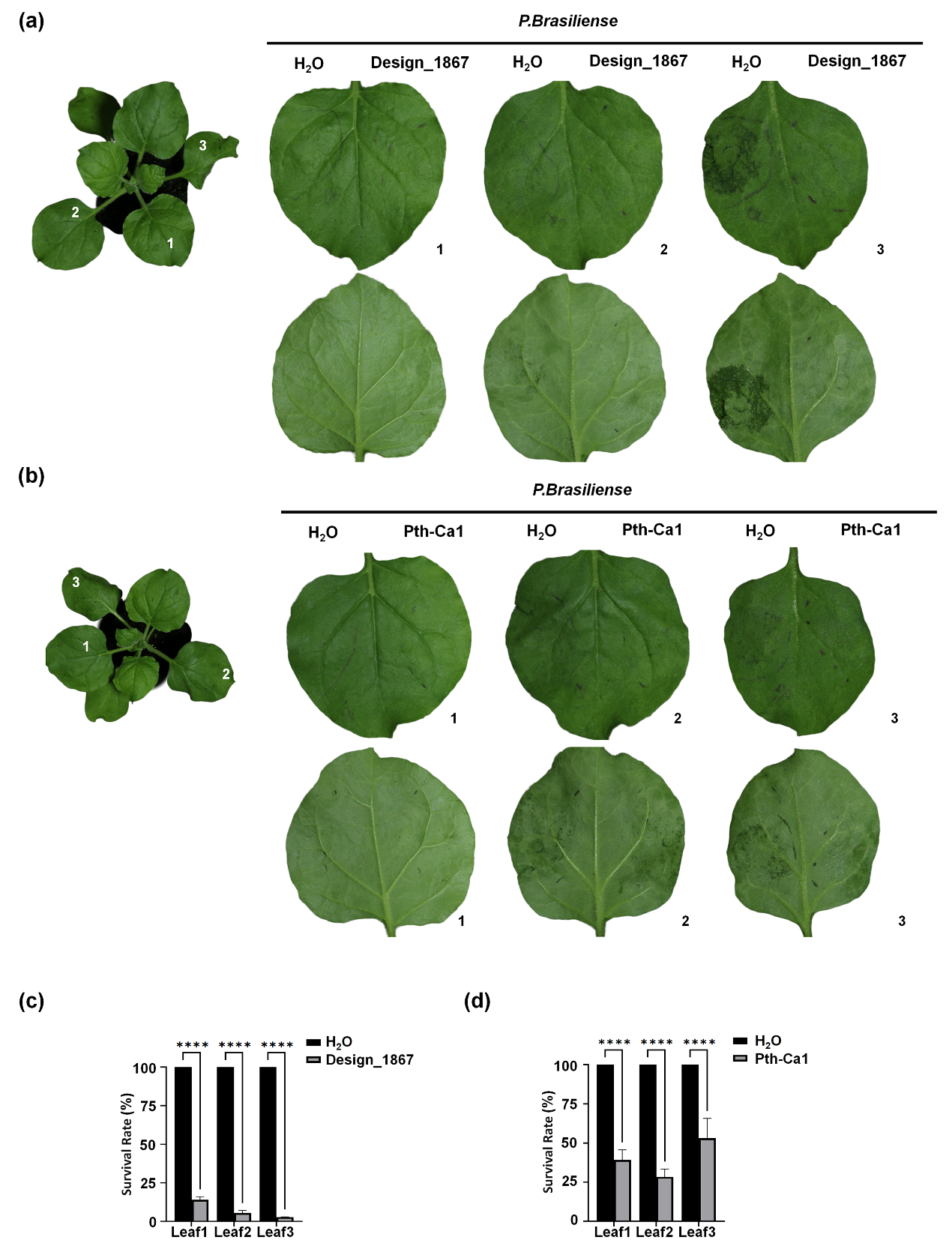


Figure S6. Comparison of the inhibitory effect of Pth-Ca1 and Design_1867 against *P. brasiliense* in *N. benthamiana*. (a) *P. brasiliense* bacterial suspension (OD₆₀₀ = 0.1) was mixed with an equal volume of Design_1867 (62.5 μg/mL) and injected into *N. benthamiana* leaves for 6 hours. No significant necrotic lesions were observed in leaves injected with Design_1867. (b) *P. brasiliense* bacterial suspension (OD₆₀₀ = 0.1) was mixed with an equal volume of Pth-Ca1 (62.5 μg/mL) and injected into *N. benthamiana* leaves for 6 hours. (c-d) Relative quantification PCR detection of *P. brasiliense* concentration in control and Design_1867/Pth-Ca1-treated leaves indicated that the bacterial load in the Design_1867-treated group was significantly lower than that in the control group (Three biological replicates were included and one representative is shown, *indicates p < 0.05, ** indicates < 0.01, *** indicates < 0.001, and ns indicates p > 0.05).
