# Supplementary material for: Protein Language Model‐Driven Optimisation of Antimicrobial Peptide Pth‐Ca1 Against Pectobacterium brasiliense Using ESMFold‐Predicted Structures and the ESM‐3 Model
Source: Mol Plant Pathol. 2026 Mar 19;27(3):e70250. doi: 10.1111/mpp.70250 (PMC13097337; doi:10.1111/mpp.70250)
Supplement: Supplementary file 7 — Figure S7: Phytotoxicity assessment of Design_1867 in plants. [file MPP-27-e70250-s004.docx]

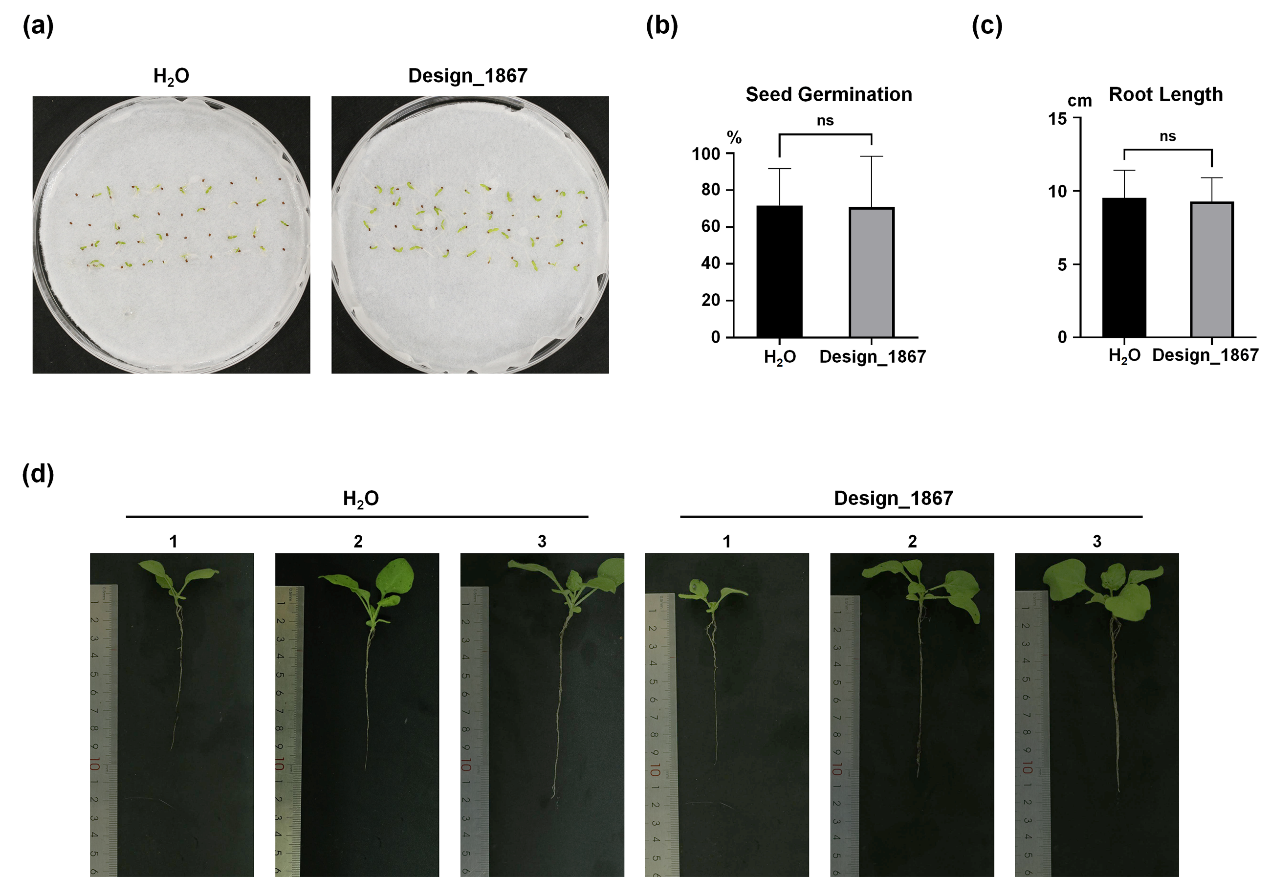


Figure S7. Phytotoxicity assessment of Design_1867 in plants. (a–b) *N. benthamiana* seeds were soaked in 2× MIC Design_1867 (62.5 μg/mL), with water used as the control, and seed germination efficiency was quantified (40 seeds per treatment, three biological replicates). The average germination rate was 72.0% for the control group and 71.0% for the Design_1867–treated group. (c–d) *N. benthamiana* seedlings were root-drenched with 2× MIC Design_1867 (62.5 μg/mL), with water as the control, and root growth was measured (n = 7 plants per treatment). The average root length was 9.53 ± 1.88 cm in the control group and 9.29 ± 1.62 cm in the Design_1867–treated group, with no significant difference observed.
