# Supplementary material for: Protein Language Model‐Driven Optimisation of Antimicrobial Peptide Pth‐Ca1 Against Pectobacterium brasiliense Using ESMFold‐Predicted Structures and the ESM‐3 Model
Source: Mol Plant Pathol. 2026 Mar 19;27(3):e70250. doi: 10.1111/mpp.70250 (PMC13097337; doi:10.1111/mpp.70250)
Supplement: Supplementary file 8 — File S1: Experimental procedures. [file MPP-27-e70250-s003.docx]

# Experimental procedures

## *Peptide Synthesis*

The peptides Pth-Ca1, Pth-St1, Design_1867, and A19 were synthesized using solid-phase chemistry by Sangon Biotech, achieving a purity of 95%.

## *De Novo Design of Pth-Ca1*

Based on Pth-Ca1, analogs were designed to retain positively charged amino acids while enhancing hydrophobic amino acids and promoting the formation of helical structures using the ESM-3 model (Hayes *et al.*, 2025). The sequence prompts used were Fixed: RK_____HR_K____RK__ (with fixed residues R, K, and H) and No_Fixed: __________________ (minimum mutants). The secondary structure prompt was specified as: HHHHHHHHHHHHHHHHHHH. Multiple temperature factors were set (0.0, 0.1, 0.2, 0.3, 0.4, and 0.5), and for each temperature factor, 1,000 peptide sequences were generated. After generation, the resulting sequences were deduplicated to ensure uniqueness. The three-dimensional structures of each peptide were predicted using ESMFold (Lin *et al.*, 2023) , and the pLDDT values along with the number of helical amino acids were calculated (Table S1). The net charge and hydrophobic ratio (%) of the peptides were determined using the Antimicrobial Peptide Database (APD3) (https://aps.unmc.edu/prediction) (Wang *et al.*, 2026) .

## *Determination of Minimum Inhibitory Concentration (MIC) and Minimum Bactericidal Concentration (MBC)*

*Escherichia coli* (*E. coli*) ATCC25922 was cultured in sterile nutrient broth (NB) medium for 6 hours and then diluted to a concentration of 1 × 10^6^ cfu/mL with sterile NB medium. The peptides were proportionally diluted with sterile water. The MIC of the peptides against *E. coli* was determined using a serial dilution method. Kanamycin at a concentration of 50 mg/mL was employed as a positive control, while sterile water served as a negative control. Cultures were incubated at 37°C for 12 hours until negative turbidity was observed; the optical density at 600 nm (OD_600_) was then measured, and results were plotted on a line graph (Wiegand *et al.*, 2008).

Subsequently, a 30 μL aliquot of the mixture was evenly spread on nutrient agar (NA) medium and incubated at 37°C for an additional 12 hours to observe the growth of *E. coli*. The concentration at which no bacterial growth was observed was considered the MBC (Bai *et al.*, 2023).

## *DNA Binding Experiments*

*E. coli* was cultured in sterile NB medium for 12 hours, then washed with sterile phosphate-buffered saline (PBS). DNA was extracted following the instructions provided in the bacterial genomic extraction kit. The concentration of the extracted DNA was measured using a Nanodrop spectrophotometer for subsequent use. To assess the interaction between peptides and DNA, 0.5 µg of DNA was mixed with varying amounts of peptides to achieve peptide/DNA mass ratios of 0, 1, 5, 10 and 50. TE buffer was added to each tube to reach a total volume of 20 µL. The mixtures were incubated in a 37°C water bath for 30 minutes. Following incubation, samples were subjected to nucleic acid gel electrophoresis, with results observed and photographed using a gel imaging system (Zhou *et al.*, 2022).

## *Scanning electron microscopy*

A total of 500 μL of *P. brasiliense* at a concentration of 1 × 10^9^ cfu/mL was mixed with 500 μL of Design_1867 at a concentration of 2 × MIC (61.25 μg/mL) and cultured at 28°C for 0 hours and 6 hours. The *P. brasiliense* cells were then fixed in a mixture containing 2.5% glutaraldehyde (in 10 mM PBS, pH 7.4) and 2% paraformaldehyde for 24 hours. Following fixation, the samples were dehydrated using a series of alcohol concentrations: 30%, 50%, 70%, 95%, and 100%, with each step lasting 15 minutes at room temperature. After critical point drying, the metal-coated samples were observed using a scanning electron microscope under 10 kV conditions (Xie *et al.*, 2021).

## *Real-time polymerase chain reaction*

The bacterial cells were collected by centrifugation at 5000 g for 5 minutes, and total RNA was extracted using Trizol reagent. Complementary DNA (cDNA) synthesis was performed using the ReverTra Ace Kit (TOYOBO, Osaka) along with random primers. Quantitative PCR (qPCR) was conducted using the TOROGreen® qPCR Master Mix Kit (TOYOBO, Osaka, Japan). The results were analyzed using the ΔΔCt method (Xie et al., 2021).

## *In Vivo Cytotoxicity Test*

To evaluate the cytocompatibility of Design_1867, HK-2 cells (renal proximal tubular cells) were seeded in 96-well plates at a density of 5×10³ cells per well and allowed to adhere overnight. The cells were then exposed to serial dilutions of Design_1867 (ranging from 0 to 500 μg/mL) in complete medium for 24 hours under standard culture conditions (37°C, 5% CO₂). Following this incubation period, 10 μL of CCK-8 reagent was added directly to each well. The plates were then incubated for an additional 2 hours at 37°C, protected from light. Absorbance at 450 nm was measured using a microplate reader, and cell viability was subsequently calculated.

*Nicotiana benthamiana* plants were cultured at 26°C under a light/dark cycle of 14 hours of light and 8 hours of darkness. The following treatments were administered via injection into the plants: sterile water, Design_1867 (61.25 μg/mL), *P. brasiliense* bacterial suspension (OD_600_ = 0.1), and *P. brasiliense* bacterial suspension (OD_600_ = 0.1) combined with Design_1867 (61.25 μg/mL). Photographs were taken 6 hours after inoculation.

## *Seed Germination Experiment*

*N. benthamiana* seeds were surface-sterilized in 75% (v/v) ethanol for 1 min and then rinsed three times with sterile water. The Design_1867 was prepared at 2× MIC (61.25 μg/mL), and an equal volume of sterile water was used as the control. For each culture dish, a sterile filter paper was placed and fully moistened with 2 mL of the Design_1867 solution or sterile water, after which 40 seeds were evenly distributed on the paper. The dishes were incubated in a growth chamber at 22 °C and 80% relative humidity under a 16 h light/8 h dark photoperiod until germination, which was defined as radicle emergence through the seed coat.

## *Root growth assay*

Germinated *N. benthamiana* seedlings were transplanted into a greenhouse and maintained under a 16 h light/8 h dark photoperiod at 22 °C with 60% relative humidity. One week after transplanting, each plant in the treatment group was treated with 15 mL of the Design_1867 at 2× MIC (61.25μg/mL), while control plants received an equal volume of water. Photographs were taken 10 days after application. Each treatment consisted of seven plants.

## *Effects of Design_1867 on the permeability of P. brasiliense cell membrane*

An overnight culture of *P. brasiliense* was prepared by inoculating log-phase cells into fresh medium at a 1:1000 dilution and incubating at 28 °C with shaking at 220 rpm. Cells were harvested by centrifugation at room temperature, washed with sterile PBS, and resuspended. The Design_1867 was then added to final concentrations of 0× MIC, 2× MIC, and 4× MIC, with 0.3% (v/v) Triton X-100 included as a positive control. Samples were incubated in a temperature-controlled water bath at 28 °C for 1 h. After incubation, samples were centrifuged at 6000 rpm at room temperature, and 100 μL of the supernatant was transferred to a 96-well plate. ONPG (o-nitrophenyl-β-D-galactopyranoside) was added to each well to a final concentration of 3 mmol/L, followed by incubation at 37 °C. Absorbance was measured at 420 nm using a microplate reader

# References

Bai, J., Li, J., Chen, Z., Bai, X., Yang, Z., Wang, Z.*, et al.* (2023) Antibacterial activity and mechanism of clove essential oil against foodborne pathogens. *LWT,* **173,** 114249.

Hayes, T., Rao, R., Akin, H., Sofroniew, N. J., Oktay, D., Lin, Z.*, et al.* (2025) Simulating 500 million years of evolution with a language model. *Science,* **387,** 850-858.

Lin, Z., Akin, H., Rao, R., Hie, B., Zhu, Z., Lu, W.*, et al.* (2023) Evolutionary-scale prediction of atomic-level protein structure with a language model. *Science,* **379,** 1123-1130.

Wang, G., Schmidt, C., Li, X. and Wang, Z. (2026) APD6: the antimicrobial peptide database is expanded to promote research and development by deploying an unprecedented information pipeline. *Nucleic Acids Res,* **54,** D363-d374.

Wiegand, I., Hilpert, K. and Hancock, R. E. (2008) Agar and broth dilution methods to determine the minimal inhibitory concentration (MIC) of antimicrobial substances. *Nat Protoc,* **3,** 163-175.

Xie, G., Gao, S., Ou, J., Zhu, M., Wu, M., Ju, X.*, et al.* (2021) Conjugating Peptides onto 1D Rodlike Bionanoparticles for Enhanced Activity against Gram-Negative Bacteria. *Nano Lett,* **21,** 1722-1728.

Zhou, L., Lian, K., Wang, M., Jing, X., Zhang, Y. and Cao, J. (2022) The antimicrobial effect of a novel peptide LL-1 on Escherichia coli by increasing membrane permeability. *BMC Microbiol,* **22,** 220.
