# Supplementary material for: Protein Language Model‐Driven Optimisation of Antimicrobial Peptide Pth‐Ca1 Against Pectobacterium brasiliense Using ESMFold‐Predicted Structures and the ESM‐3 Model
Source: Mol Plant Pathol. 2026 Mar 19;27(3):e70250. doi: 10.1111/mpp.70250 (PMC13097337; doi:10.1111/mpp.70250)
Supplement: Supplementary file 9 — Table S1: Homologous genes of Pth‐St1. [file MPP-27-e70250-s007.docx]

**Table. S1** **Homologous genes of Pth-St1**

| Gene_ID | Sequences | Species | Positive  Charge | Negative  Charge | Net Charge | Hydrophobic ratio |
| --- | --- | --- | --- | --- | --- | --- |
| >AAB31351.1:1-19 | RNCESLSHRFKGPCTRDSN | Solanum_tuberosum | 5 | 2 | 3 | 21.05263158 |
| >AAL15885.1:32-50 | RTCESQSHRFKGPCVRKSN | Castanea_sativa | 6 | 1 | 5 | 21.05263158 |
| >AIT42149.1:32-50 | RHCESLSHRFKGPCASDRN | Solanum_tuberosum | 6 | 2 | 4 | 26.31578947 |
| >AKQ53348.1:32-50 | RTCESQSHRFKGPCSRDSN | Nicotiana_benthamiana | 5 | 2 | 3 | 15.78947368 |
| >ALJ76367.1:32-50 | RTCESQSHRFKGPCSRDSN | synthetic construct | 5 | 2 | 3 | 15.78947368 |
| >AOD75394.1:32-50 | RTCESQSHRFKGPCARDSN | Nicotiana_alata | 5 | 2 | 3 | 21.05263158 |
| >CAD6226261.1:32-50 | RKCESQSHRFKGPCSRDAN | Miscanthus_lutarioriparius | 6 | 2 | 4 | 21.05263158 |
| >CAH8266493.1:9-27 | RTCESQSHRFKGTCVRESN | Arabidopsis_lyrata | 5 | 2 | 3 | 21.05263158 |
| >CAL5076240.1:32-50 | RDCQSPSHRYKGPCVRDSN | Urochloa_decumbens | 5 | 2 | 3 | 15.78947368 |
| >CDP10468.1:32-50 | RTCESLSHRFKGTCVRGSN | Coffea_canephora | 5 | 1 | 4 | 26.31578947 |
| >GFQ06714.1:28-46 | RTCESLSHRFKGPCGRDSN | Phtheirospermum_japonicum | 5 | 2 | 3 | 21.05263158 |
| >KAA0874854.1:28-46 | RTCESKSHRFKGPCVRDSN | Enterobacter_hormaechei | 6 | 2 | 4 | 21.05263158 |
| >KAF0911053.1:34-52 | RTCESQSHRFKGPCVRKSN | Oryza_meyeriana_var._granulata | 6 | 1 | 5 | 21.05263158 |
| >KAF6174640.1:29-47 | RTCESASQRFKGPCGRDSN | Kingdonia_uniflora | 4 | 2 | 2 | 21.05263158 |
| >KAG5594094.1:32-50 | RHCESLSHRFKGPCSSDRN | Solanum_commersonii | 6 | 2 | 4 | 21.05263158 |
| >KAJ0238012.1:31-49 | RTCESQSHRFKGPCVSESN | Hirschfeldia_incana | 4 | 2 | 2 | 21.05263158 |
| >KAJ3689728.1:64-82 | RTCESQSHKFKGPCVRQSN | Hirschfeldia_incana | 5 | 1 | 4 | 21.05263158 |
| >KAK4597081.1:31-49 | RTCESQSHRFKGPCVRKSN | Rhynchospora_tenuis | 6 | 1 | 5 | 21.05263158 |
| >KAK4713047.1:32-50 | RNCESLSHRFKGPCASDKN | Solanum_pinnatisectum | 5 | 2 | 3 | 26.31578947 |
| >KAK7346138.1:62-80 | RTCESQSHRFKGPCVSDTN | Phaseolus_coccineus | 4 | 2 | 2 | 21.05263158 |
| >KAK9117956.1:33-51 | RTCESASHKFKGPCGRDSN | Stephania_cephalantha | 5 | 2 | 3 | 21.05263158 |
| >KAL0827478.1:31-49 | RTCESQSHRFKGPCVSDNN | Brassica_carinata | 4 | 2 | 2 | 21.05263158 |
| >KAL3652818.1:28-46 | RLCESKSNRFKGPCARDSN | Castilleja_foliolosa | 5 | 2 | 3 | 26.31578947 |
| >KDP32807.1:31-49 | RTCESLSHRFKGICVRNSN | Jatropha_curcas | 5 | 1 | 4 | 26.31578947 |
| >MCD7471483.1:27-45 | RTCESQSHRFKGPCVRKSN | Datura_stramonium | 6 | 1 | 5 | 21.05263158 |
| >MED6119135.1:32-50 | RTCESQSHRFKGPCVSDTN | Stylosanthes_scabra | 4 | 2 | 2 | 21.05263158 |
| >MQM10943.1:34-52 | RTCESQSHRFKGPCFRASN | Colocasia_esculenta | 5 | 1 | 4 | 26.31578947 |
| >NP_001152925.1:32-50 | RKCESQSFRFKGPCSRDAN | Zea_mays | 5 | 2 | 3 | 26.31578947 |
| >NP_001295621.1:31-49 | RTCESLSHRFKGICVRNSN | Jatropha_curcas | 5 | 1 | 4 | 26.31578947 |
| >NP_001333453.1:32-50 | RHCESLSHRFKGPCVSDKN | Solanum_lycopersicum | 6 | 2 | 4 | 26.31578947 |
| >NP_001404100.1:34-5 | RTCESQSHRFKGPCARKAN | Oryza_sativa_Japonica_Group | 6 | 1 | 5 | 26.31578947 |
| >PON78327.1:31-49 | RTCESQSHRFKGPCVRKSN | Parasponia_andersonii | 6 | 1 | 5 | 21.05263158 |
| >RDY09218.1:31-49 | RTCESQSHRFKGPCVSDTN | Mucuna_pruriens | 4 | 2 | 2 | 21.05263158 |
| >RLN32874.1:28-46 | RECQSPSHRFKGPCARDAN | Panicum_miliaceum | 5 | 2 | 3 | 26.31578947 |
| >TMW80959.1:33-51 | RTCESQSHSFKGPCSRDSN | Solanum_chilense | 4 | 2 | 2 | 15.78947368 |
| >TMW83265.1:31-49 | RHCESLSHRFKGPCVSDKN | Solanum_chilense | 6 | 2 | 4 | 26.31578947 |
| >URN71264.1:10-28 | RTCESQSHRFKGPCVSDTN | Phaseolus_vulgaris | 4 | 2 | 2 | 21.05263158 |
| >WVZ25187.1:33-51 | RTCESQSHRFKGPCVSDTN | Vigna_mungo | 4 | 2 | 2 | 21.05263158 |
| >XP_002459603.1:33-51 | RKCESPSHRFQGPCSRDAN | Sorghum_bicolor | 5 | 2 | 3 | 21.05263158 |
| >XP_003630421.1:28-46 | RRCESKSHKFKGPCSRDSN | Medicago_truncatula | 7 | 2 | 5 | 15.78947368 |
| >XP_009593293.1:32-50 | RTCESQSHRFKGPCSRDSN | Nicotiana_tomentosiformis | 5 | 2 | 3 | 15.78947368 |
| >XP_009769843.1:32-50 | RTCESQSHRFKGPCSRDSN | Nicotiana_sylvestris | 5 | 2 | 3 | 15.78947368 |
| >XP_013618268.1:31-49 | RTCESQSHRFKGPCVSDNN | Brassica_oleracea_var._oleracea | 4 | 2 | 2 | 21.05263158 |
| >XP_013691870.2:31-49 | RTCESQSHRFKGPCVSDNN | Brassica_napus | 4 | 2 | 2 | 21.05263158 |
| >XP_015080770.1:32-50 | RHCESLSHRFKGPCASDKN | Solanum_pennellii | 6 | 2 | 4 | 26.31578947 |
| >XP_016451209.1:32-50 | RTCESQSHRFKGPCSRDSN | Nicotiana_tabacum | 5 | 2 | 3 | 15.78947368 |
| >XP_017428548.1:32-50 | RTCESQSHRFKGPCVSDTN | Vigna_angularis | 4 | 2 | 2 | 21.05263158 |
| >XP_019224135.1:32-50 | RTCESQSHRFKGPCSRDSN | Nicotiana_attenuata | 5 | 2 | 3 | 15.78947368 |
| >XP_020085638.1:32-50 | RTCEAQSHRFKGPCVRASN | Ananas_comosus | 5 | 1 | 4 | 31.57894737 |
| >XP_020229972.1:32-50 | RTCESQSHRFKGPCVSDTN | Cajanus_cajan | 4 | 2 | 2 | 21.05263158 |
| >XP_020260248.1:32-50 | RTCESQSHRFRGPCVRESN | Asparagus_officinalis | 5 | 2 | 3 | 21.05263158 |
| >XP_022846608.1:32-50 | RICESLSHRFKGPCVRNAN | Olea_europaea_var._sylvestris | 5 | 1 | 4 | 31.57894737 |
| >XP_027916869.1:32-50 | RTCESQSHRFKGPCVSDTN | Vigna_unguiculata | 4 | 2 | 2 | 21.05263158 |
| >XP_030512858.1:31-49 | RTCESQSHRFKGACVRDSN | Rhodamnia_argentea | 5 | 2 | 3 | 26.31578947 |
| >XP_030960725.1:31-49 | RTCESQSHRFKGPCVRKSN | Quercus_lobata | 6 | 1 | 5 | 21.05263158 |
| >XP_045823366.1:28-46 | RRCESKSHKFKGPCARDSN | Trifolium_pratense | 7 | 2 | 5 | 21.05263158 |
| >XP_047159190.1:32-50 | RTCESQSHRFKGPCVSDTN | Vigna_umbellata | 4 | 2 | 2 | 21.05263158 |
| >XP_047268587.1:27-45 | RKCESQSHRFKGPCVRKSN | Capsicum_annuum | 7 | 1 | 6 | 21.05263158 |
| >XP_049359691.1:32-50 | RNCESLSHRFKGPCVSDKN | Solanum_verrucosum | 5 | 2 | 3 | 26.31578947 |
| >XP_049377408.1:32-50 | RHCESLSHRFKGPCASDKN | Solanum_stenotomum | 6 | 2 | 4 | 26.31578947 |
| >XP_054809759.1:31-49 | RTCESQSHRFKGPCIRKSN | Prosopis_cineraria | 6 | 1 | 5 | 15.78947368 |
| >XP_057420574.1:32-50 | RDCESQSHRFKGPCVSDTN | Prosopis_cineraria | 4 | 3 | 1 | 21.05263158 |
| >XP_058098101.1:59-77 | RTCESQSHRFKGPCVRGSN | Magnolia_sinica | 5 | 1 | 4 | 21.05263158 |
| >XP_062113099.1:31-49 | RTCEAQSHRFKGPCVRKSN | Humulus_lupulus | 6 | 1 | 5 | 26.31578947 |
| >XP_068465986.1:32-50 | RTCESQSHRFKGPCVSDTN | Phaseolus_vulgaris | 4 | 2 | 2 | 21.05263158 |
| >XP_070055743.1:9-27 | RTCESQSHRFKGPCVRKSN | Nicotiana_tomentosiformis | 6 | 1 | 5 | 21.05263158 |
