# Supplementary material for: Protein Language Model‐Driven Optimisation of Antimicrobial Peptide Pth‐Ca1 Against Pectobacterium brasiliense Using ESMFold‐Predicted Structures and the ESM‐3 Model
Source: Mol Plant Pathol. 2026 Mar 19;27(3):e70250. doi: 10.1111/mpp.70250 (PMC13097337; doi:10.1111/mpp.70250)
Supplement: Supplementary file 10 — Table S2: Analysis of fixed and No_Fixed mutations in Pth‐Ca1. [file MPP-27-e70250-s002.docx]

**Table. S2** **Analysis of Fixed and No_Fixed Mutations in Pth-Ca1**

| **Model** | **ID** | **Sequence** | **Mutation Count** | **pLDDT** | **Helix Residues** | **Net Charge** | **Hydrophobicratio%** |
| --- | --- | --- | --- | --- | --- | --- | --- |
| No_Fixed:  Minimum mutation | design_2916 | RKQVSQSLRELGDCVRKSN | 6 | 88.687368 | 17 | +3 | 26 |
|  | design_1048 | RLCESLSHLFKGLLVRLSN | 6 | 89.036316 | 17 | +2.25 | 47 |
|  | design_3733 | RKLESLSHRFLGELVRKSN | 5 | 87.699474 | 17 | +3.25 | 32 |
|  | design_2831 | RKRLSQSLRFLGRCVRKSN | 5 | 87.928421 | 17 | +7 | 32 |
|  | design_2899 | RKKLSQSLRFLGDQVRKSN | 6 | 89.455263 | 17 | +5 | 26 |
|  | design_1937 | RKFEIQSHRFKKLCVKLSN | 6 | 88.830000 | 17 | +5.25 | 37 |
|  | design_4628 | RELESQVHRFIGECVRISN | 6 | 89.725263 | 17 | +0.25 | 37 |
|  | design_2906 | RELESQLHRLKGLCVRLSN | 6 | 89.636316 | 17 | +2.25 | 37 |
|  | design_2855 | RELESQLHRFKGECVRKLL | 6 | 88.481053 | 17 | +2.25 | 37 |
|  | design_34 | RKLLSQYRRFKGACVRLSN | 6 | 87.918421 | 17 | +6 | 37 |
|  | design_4644 | RQLESLSHRFKGLCDRLSN | 6 | 88.190000 | 17 | +2.25 | 32 |
|  | design_1036 | RKCEELSHLFKGLLVRKLN | 6 | 88.184737 | 17 | +3.25 | 42 |
|  | design_2916 | RKQVSQSLRELGDCVRKSN | 6 | 88.687368 | 17 | +3 | 26 |
| Fixed:  Fixed R, H, K | design_2552 | RKKERQKHRKKKRKKRKSN | 8 | 90.277368 | 17 | +13.25 | 0 |
|  | design_4260 | RKKERESHRKKEREVRKAN | 8 | 90.777368 | 17 | +6.25 | 11 |
|  | design_1867 | RKLVRQLHRFKGKLVRKLH | 8 | 90.817895 | 17 | +8.5 | 37 |
|  | design_3212 | RKAELASHRFKAACVRKAL | 7 | 90.926842 | 17 | +5.25 | 53 |
|  | design_2519 | RKKERKSHRKKEKKKRKSN | 8 | 90.608421 | 17 | +11.25 | 0 |
|  | design_1760 | RKLKRKLHRFKGKLVRKLN | 8 | 90.014211 | 17 | +10.25 | 32 |
|  | design_1551 | RKLESEIHRLKGECVRKSR | 6 | 90.249474 | 17 | +4.25 | 26 |
|  | design_1216 | RKLLRKLHRFKGKLVRKLN | 8 | 90.516316 | 17 | +9.25 | 37 |
|  | design_2562 | RKKERQKHRKKERKVRKKN | 8 | 90.983158 | 17 | +11.25 | 5 |
|  | design_3240 | RKLQKQLHRFKGKLVRKLN | 7 | 90.031053 | 17 | +8.25 | 32 |
|  | design_311 | RKSERKSHRRKRKCERKSH | 8 | 90.135263 | 17 | +9.5 | 5 |
|  | design_306 | RKVEEALHRFKGKLVRKLK | 8 | 90.164737 | 17 | +6.25 | 37 |
|  | design_1807 | RKKERQSHRKKERQVRKKE | 8 | 90.204737 | 17 | +8.25 | 5 |
