# Supplementary material for: Protein Language Model‐Driven Optimisation of Antimicrobial Peptide Pth‐Ca1 Against Pectobacterium brasiliense Using ESMFold‐Predicted Structures and the ESM‐3 Model
Source: Mol Plant Pathol. 2026 Mar 19;27(3):e70250. doi: 10.1111/mpp.70250 (PMC13097337; doi:10.1111/mpp.70250)
Supplement: Supplementary file 11 — Table S3: Structural comparison between ESM‐3 generated structures and ESMFold predictions. [file MPP-27-e70250-s015.docx]

**Table S3. Structural comparison between ESM-3 generated structures and ESMFold predictions**

| **Strategy** | **ID** | **RMSD (Å)** | **TM-score** |
| --- | --- | --- | --- |
| Fixed | design_1216 | 0.57 | 0.736 |
| Fixed | design_1551 | 0.42 | 0.705 |
| Fixed | design_1760 | 0.59 | 0.681 |
| Fixed | design_1807 | 0.38 | 0.805 |
| Fixed | design_1867 | 0.43 | 0.712 |
| Fixed | design_2519 | 0.32 | 0.753 |
| Fixed | design_2552 | 0.46 | 0.764 |
| Fixed | design_2562 | 0.38 | 0.797 |
| Fixed | design_306 | 0.35 | 0.674 |
| Fixed | design_311 | 0.68 | 0.655 |
| Fixed | design_3212 | 0.36 | 0.669 |
| Fixed | design_3240 | 0.48 | 0.730 |
| Fixed | design_4260 | 0.47 | 0.691 |
| No_Fixed | design_1036 | 0.59 | 0.630 |
| No_Fixed | design_1048 | 0.56 | 0.686 |
| No_Fixed | design_1937 | 1.66 | 0.231 |
| No_Fixed | design_2831 | 0.44 | 0.652 |
| No_Fixed | design_2855 | 0.66 | 0.658 |
| No_Fixed | design_2899 | 0.42 | 0.709 |
| No_Fixed | design_2906 | 0.37 | 0.742 |
| No_Fixed | design_2916 | 0.38 | 0.716 |
| No_Fixed | design_34 | 0.63 | 0.646 |
| No_Fixed | design_3733 | 0.8 | 0.610 |
| No_Fixed | design_4628 | 0.33 | 0.707 |
| No_Fixed | design_4644 | 0.58 | 0.707 |
