# Supplementary material for: Protein Language Model‐Driven Optimisation of Antimicrobial Peptide Pth‐Ca1 Against Pectobacterium brasiliense Using ESMFold‐Predicted Structures and the ESM‐3 Model
Source: Mol Plant Pathol. 2026 Mar 19;27(3):e70250. doi: 10.1111/mpp.70250 (PMC13097337; doi:10.1111/mpp.70250)
Supplement: Supplementary file 12 — Table S4: Characterisation and antimicrobial activity of Pth‐Ca1 and its derivatives. [file MPP-27-e70250-s014.docx]

**Table.S4** **Characterization and Antimicrobial Activity of Pth-Ca1 and Its Derivatives**

| **Name** | **Sequences** | **helix** | **Hyd** | **HMom** | **z** | **FreqPolar** | **FreqNonPolar** | **Hydrophobic**  **face** | **Hydrophilic**  **face** | **Clump Count** |
| --- | --- | --- | --- | --- | --- | --- | --- | --- | --- | --- |
| Pth-Ca1 | RKCESQSHRFKGPCVRKSN | 1 | -0.034 | 0.331 | 5 | 0.737 | 0.263 | \ | \ | >500 |
| Pth-St1 | RNCESLSHRFKGPCTRDSN | 1 | 0.049 | 0.411 | 2 | 0.737 | 0.263 | PL | \ | \ |
| Design_1867 | RKLVRQLHRFKGKLVRKLH | 1 | 0.162 | 0.613 | 8 | 0.632 | 0.368 | FLLLL | GRR | 2.67±0.58 |
| Design_3240 | RKLQKQLHRFKGKLVRKLN | 1 | 0.048 | 0.593 | 8 | 0.684 | 0.316 | FLLLL | GKR | 76.40±16.01 |
| Design_34 | RKLLSQYRRFKGACVRLSN | 1 | 0.211 | 0.515 | 6 | 0.579 | 0.421 | LFL | \ | 12.33±10.12 |
| Design_1937 | RKFEIQSHRFKKLCVKLSN | 1 | 0.218 | 0.424 | 5 | 0.632 | 0.368 | LFF | \ | 74.33±16.50 |
| Design_306 | RKVEEALHRFKGKLVRKLK | 1 | 0.027 | 0.555 | 6 | 0.632 | 0.368 | FVLLL | GER | >200 |
| Design_1216 | RKLLRKLHRFKGKLVRKLN | 1 | 0.108 | 0.655 | 9 | 0.632 | 0.368 | FLLLL | GRR | 10.67±2.31 |
| Design_2831 | RKRLSQSLRFLGRCVRKSN | 1 | 0.088 | 0.494 | 7 | 0.684 | 0.316 | LLVL | \ | >200 |
| Design_1760 | RKLKRKLHRFKGKLVRKLN | 1 | -0.034 | 0.58 | 10 | 0.684 | 0.316 | FLLLL | GRR | 18.33±3.06 |

Hyd: Calculation of the mean hydrophobicity

HMom: Calculation of the mean amphipathic moment

z: Calculation of the net charge

FreqPolar: polar residues ratio

FreqNonPolar: Non polar residues ratio

Clump Count: The spread plate method was used to enumerate bacterial colonies and assay peptide activity.
