# Supplementary material for: Protein Language Model‐Driven Optimisation of Antimicrobial Peptide Pth‐Ca1 Against Pectobacterium brasiliense Using ESMFold‐Predicted Structures and the ESM‐3 Model
Source: Mol Plant Pathol. 2026 Mar 19;27(3):e70250. doi: 10.1111/mpp.70250 (PMC13097337; doi:10.1111/mpp.70250)
Supplement: Supplementary file 13 — Table S5: Primers used in this study. [file MPP-27-e70250-s011.docx]

**Table. S5** **Primers used in this study**

| **Gene function** | **Primer Names** | **Sequence (5’-3’)** |
| --- | --- | --- |
| outer membrane porin F | ompF f | GACAGCAGCGTCACTGATAC |
|  | ompF r | GCAGCATTACCACCATCGTT |
| a reactive oxygen species-related gene | robA f | TCCGCGCTCGGATATATGAA |
|  | robA r | GGAAAGCCATCTCGACCAAC |
| involved in the translocation of LPS from the inner membrane to the outer membrane | lptF f | CGCCACCATTAACGTCATGT |
|  | lptF r | CCAGCACTTCTTCCTGATGC |
| a biofilm-related gene | bolA f | TGATGAGAGCTATCGCCACAA |
|  | bolA r | CGCTGACCAGCACAACTTTA |
| internal reference gene | 16S rRNA f | TGATAAACCGGAGGAAGGTG |
|  | 16S rRNA r | TTCATGGAGTCGAGTTGCAG |
