# Supplementary material for: Protein Language Model‐Driven Optimisation of Antimicrobial Peptide Pth‐Ca1 Against Pectobacterium brasiliense Using ESMFold‐Predicted Structures and the ESM‐3 Model
Source: Mol Plant Pathol. 2026 Mar 19;27(3):e70250. doi: 10.1111/mpp.70250 (PMC13097337; doi:10.1111/mpp.70250)
Supplement: Supplementary file 14 — Table S6: Cytotoxicity of the designed peptide 1867. [file MPP-27-e70250-s009.docx]

**Table. S6** **Cytotoxicity of the Designed Peptide 1867**

|  | OD_450_ | | | | | | | | |
| --- | --- | --- | --- | --- | --- | --- | --- | --- | --- |
| Design_1867 | 0 μg/mL | 0.1 μg/mL | 1 μg/mL | 5 μg/mL | 10 μg/mL | 50 μg/mL | 100 μg/mL | 200 μg/mL | 500 μg/mL |
| Repeat 1 | 0.4560 | 0.4700 | 0.5030 | 0.4880 | 0.4662 | 0.5132 | 0.5389 | 0.5192 | 0.5408 |
| Repeat 2 | 0.5277 | 0.4897 | 0.5607 | 0.5425 | 0.4886 | 0.5206 | 0.5728 | 0.5862 | 0.6119 |
| Repeat 3 | 0.5555 | 0.5554 | 0.5242 | 0.5022 | 0.4945 | 0.5155 | 0.5673 | 0.5059 | 0.5552 |
| Repeat 4 | 0.5355 | 0.4729 | 0.4877 | 0.5243 | 0.5164 | 0.5848 | 0.5618 | 0.5192 | 0.5592 |
| Repeat 5 | 0.5513 | 0.5452 | 0.5051 | 0.5112 | 0.5273 | 0.5403 | 0.5479 | 0.5996 | 0.5481 |
| Repeat 6 | 0.5478 | 0.4573 | 0.5242 | 0.5122 | 0.4606 | 0.5578 | 0.6531 | 0.5042 | 0.5614 |
