# Supplementary material for: Protein Language Model‐Driven Optimisation of Antimicrobial Peptide Pth‐Ca1 Against Pectobacterium brasiliense Using ESMFold‐Predicted Structures and the ESM‐3 Model
Source: Mol Plant Pathol. 2026 Mar 19;27(3):e70250. doi: 10.1111/mpp.70250 (PMC13097337; doi:10.1111/mpp.70250)
Supplement: Supplementary file 15 — Table S7: Calculation of the discriminant factor D. [file MPP-27-e70250-s006.docx]

**Table. S7 Calculation of the discriminant factor D**

| **Name** | **Sequences** | **HMom** | **z** | **Helix ratio (%)** | **D Value** | **Class** |
| --- | --- | --- | --- | --- | --- | --- |
| Pth-Ca1 | RKCESQSHRFKGPCVRKSN | 0.331 | 5 | 0.416666667 | 1.962464 | / |
| Pth-St1 | RNCESLSHRFKGPCTRDSN | 0.411 | 2 | 0.25 | 1.047984 | / |
| Design_1867 | RKLVRQLHRFKGKLVRKLH | 0.613 | 8 | 1 | 3.218672 | Lipid-Binding Helix |
| Design_3240 | RKLQKQLHRFKGKLVRKLN | 0.593 | 8 | 1 | 3.199792 | Lipid-Binding Helix |
| Design_34 | RKLLSQYRRFKGACVRLSN | 0.515 | 6 | 1 | 2.46616 | Lipid-Binding Helix |
| Design_1937 | RKFEIQSHRFKKLCVKLSN | 0.424 | 5 | 1 | 2.050256 | Lipid-Binding Helix |
| Design_306 | RKVEEALHRFKGKLVRKLK | 0.555 | 6 | 1 | 2.50392 | Lipid-Binding Helix |
| Design_1216 | RKLLRKLHRFKGKLVRKLN | 0.655 | 9 | 1 | 3.58832 | Lipid-Binding Helix |
| Design_2831 | RKRLSQSLRFLGRCVRKSN | 0.494 | 7 | 1 | 2.776336 | Lipid-Binding Helix |
| Design_1760 | RKLKRKLHRFKGKLVRKLN | 0.58 | 10 | 1 | 3.84752 | Lipid-Binding Helix |
